# Sensitivity of Vegetation Indices for Estimating Vegetative N Status in Winter Wheat

**DOI:** 10.3390/s19173712

**Published:** 2019-08-27

**Authors:** Lukas Prey, Urs Schmidhalter

**Affiliations:** Chair of Plant Nutrition, Department of Plant Sciences, Technical University of Munich, 85354 Freising, Germany

**Keywords:** precision farming, phenomics, nitrogen fertilization, red edge, hyperspectral proximal sensing, nitrogen use efficiency, nitrogen uptake, nitrogen concentration, nitrogen nutrition index, noise equivalent, smart farming

## Abstract

Precise sensor-based non-destructive estimation of crop nitrogen (N) status is essential for low-cost, objective optimization of N fertilization, as well as for early estimation of yield potential and N use efficiency. Several studies assessed the performance of spectral vegetation indices (SVI) for winter wheat (*Triticum aestivum* L.), often either for conditions of low N status or across a wide range of the target traits N uptake (Nup), N concentration (NC), dry matter biomass (DM), and N nutrition index (NNI). This study aimed at a critical assessment of the estimation ability depending on the level of the target traits. It included seven years’ data with nine measurement dates from early stem elongation until flowering in eight N regimes (0–420 kg N ha^−1^) for selected SVIs. Tested across years, a pronounced date-specific clustering was found particularly for DM and NC. While for DM, only the R900_970 gave moderate but saturated relationships (R^2^ = 0.47, *p* < 0.001) and no index was useful for NC across dates, NNI and Nup could be better estimated (REIP: R^2^ = 0.59, *p* < 0.001 for both traits). Tested within growth stages across N levels, the order of the estimation of the traits was mostly Nup ≈ NNI > NC ≈ DM. Depending on the number (n = 1–3) and characteristic of cultivars included, the relationships improved when testing within instead of across cultivars, with the relatively lowest cultivar effect on the estimation of DM and the strongest on NC. For assessing the trait estimation under conditions of high–excessive N fertilization, the range of the target traits was divided into two intervals with NNI values < 0.8 (interval 1: low N status) and with NNI values > 0.8 (interval 2: high N status). Although better estimations were found in interval 1, useful relationships were also obtained in interval 2 from the best indices (DM: R780_740: average R^2^ = 0.35, RMSE = 567 kg ha^−1^; NC: REIP: average R^2^ = 0.40, RMSE = 0.25%; NNI: REIP: average R^2^ = 0.46, RMSE = 0.10; Nup: REIP: average R^2^ = 0.48, RMSE = 21 kg N ha^−1^). While in interval 1, all indices performed rather similarly, the three red edge-based indices were clearly better suited for the three N-related traits. The results are promising for applying SVIs also under conditions of high N status, aiming at detecting and avoiding excessive N use. While in canopies of lower N status, the use of simple NIR/VIS indices may be sufficient without losing much precision, the red edge information appears crucial for conditions of higher N status. These findings can be transferred to the configuration and use of simpler multispectral sensors under conditions of contrasting N status in precision farming.

## 1. Introduction

Optimizing nitrogen (N) supply in wheat production is essential, both economically and for reducing environmental side effects from excessive N supply [1,2,3,4]. Unlike under West European conditions, optimum N fertilization levels are often largely exceeded in China [5,6,7] where only about 30% of the applied N is removed through the produce [8]. In contrast, the prevailing N intensities are below the optimum N input in many East European and African regions [9]. Therefore, estimating the N nutrition status is crucial for in-season optimization of the N fertilization under varying conditions. Currently, the in-season estimation of the N-status is still focused on optimizing/maximizing grain yields. However, the reduction of N surplus and deficits are equally important but require the detection of over-fertilization and N deficits, respectively [10].

Low-throughput measurements with the Soil Plant Analysis Development (SPAD) sensors were found to be useful for detecting N deficiency in relation to sufficiently fertilized treatments. The SPAD was used to estimate the N status [11,12] and to recommend optimum fertilization levels at high N status [7]. However, due to being time-consuming, covering only a small spot of selected leaves and saturating values, SPAD measurements may not be suitable for detecting over-fertilization [7,11]. Moreover, SPAD measurements are restricted to estimating the leaf chlorophyll and the commonly closely related N concentration (NC), but neglect the biomass. Instead, the nitrogen nutrition index (NNI) is used for quantifying N deficiency through relating the actual NC to a critical NC value, which depends on the biomass. NNI values < 1 correspond to a sub-optimal N status whereas NNI > 1 indicates excessive N supply [13,14,15,16,17,18].

Non-destructive, spectral sensing has been established for estimating the N status, including dry matter weight (DM), NC, Nup and the NNI [10,17,19,20,21,22,23,24]. The red edge inflection point (REIP) was recommended for the estimation of the NNI [15,25].) Slightly better relationships for the NNI than for Nup were found [15]. These authors reported a shift in the index ~ NNI relationship between years and growth stages, possibly attributed to differing N allocation, differing sensor calibrations or cultivar effects. Other authors confirmed REIP-related red edge indices for direct estimation of the N status with active and passive sensors [22,23,26,27]. Alternatively, a ‘mechanistic’ approach for NNI estimation through optimized estimations of the components DM and NC was found to be less affected by the phenology [25], but requires separate calibration efforts.

Several studies indicated a better detection of N traits than that of DM [22,28,29,30]. Red edge information is most suitable for estimating N status and was found to be superior over classical NIR/red indices such as the NDVI for DM for overcoming saturation [31]. The water band index was often suited for biomass estimation during the generative phase and may be used also at pre-flowering [22]. Simple ratio indices such as the R780_840 [30] and the R760_730 [22] were found to perform similarly or better than the REIP for N-related traits.

Persisting limitations occur from the differing index rankings by growth stages [15,22,32] as well as from the limited transferability between growth stages. A stronger seasonal shift was found for Nup than for NNI from index relationships compared within and across the stem elongation and booting stage [23]. Still, the indirect NNI estimation via models optimized for DM and NC was recommended for reducing the effect of phenology [25]. Moreover, the index use is affected by total above-ground DM [28]. Substantial differences in the index sensitivity especially beyond an Nup of approximately 150 kg N ha^−1^ and for NC beyond 2% were found [28]. Therefore, it is conceivable that required sensor specifications differ for applications of low, medium and high-excessive N status, corresponding for example to conditions in parts of America and East Europe, Germany (with common N applications of ≈180 kg N ha^−1^) and China (with frequent N applications > 300 kg N ha^−1^; [33]), respectively. While the use of NDVI-based sensors also under Chinese conditions resulted in considerable reductions in N use [33], further comparisons indicated that red edge information can substantially improve the estimation of the N status under high N conditions [22,26]. In spite of differing growing conditions, it was possible to transfer index-based models between different growing regions [28,33].

Some studies proposed further optimized index equations. However, the transferability to multispectral sensors is often limited by differing spectral configurations.

Moreover, in many cases, the index comparison was conducted only over a wide data range of the reference traits created by N levels beyond 300 kg N ha^−1^ and often included non-N fertilized plots [23,25,26,34]. In contrast, the optimum N level under Chinese conditions is often below 200 kg N ha^−1^ [5,7]. This may have resulted into a too optimistic appraisal on the achieved accuracy without discussing the level and range of the target traits, which are relevant in practice. The latter are however crucial for estimating the N status in high-yielding environments and for potentially identifying effects of over-fertilization within a limited data range [35]. In addition, the disturbing effect of cultivar-specific morphology and phenology can affect the use of spectral methods [28]. However, the effect was rarely compared, while mostly the relationships were tested either for one cultivar or across cultivars. Comparing index relationships both within and across morphologically differing German and Chinese cultivars, often weaker relationships were observed across cultivar groups [28]. Due to the complex effects, further empirical testing under varying conditions is required.

Using seven years’ data of an N fertilization trial conducted in high-yielding West-European conditions, this study aimed at comparing selected vegetation indices for estimating wheat traits related to the vegetative and pre-flowering canopy N status. Traits included NNI, Nup and their contributing traits DM and NC. The objective of the study was to investigate empirically the index rankings and the trait estimability as influenced by (i) years and growth stages, (ii) cultivars within years and (iii) by the N status and the absolute level of the target traits.

## 2. Materials and Methods

### 2.1. Field Trials

The experiment was conducted over seven years in 2009–2011, 2013, 2014, 2016, and 2018 in southeast Germany (48.406 N, 11.692 E) under rain-fed conditions to evaluate the N response in biomass and nitrogen accumulation, and sensor-based detection of over-fertilization. The soil was mostly homogeneous Cambisol of silty clay loam with a pH of 6.4, K_2_O-content of 12 mg 100 g^−1^, P_2_O_5_-content of 12 mg 100 g^−1^ and C_org_ of 1.2%. The annual precipitation in this region is approximately 800 mm with an average temperature of 8 °C. In all years, N fertilization was differentiated in eight N levels, ranging from 0 to 420 kg N ha^−1^, incrementally increasing by 60 kg N ha^−1^. According to the local practice, N application was split into three doses. N was applied at the beginning of vegetation in spring, at early stem elongation, and at booting-flowering (Table 1). N fertilizer was urea (2009–2013) ammonium nitrate (2015–2018) or a combination of both fertilizers (in 2014). The trial was set up in four replicates and consisted of differing numbers of cultivars (3 in 2009, 1 in 2010, 3 in 2011, 1 in 2013, 1 in 2014, and 2 in 2016 and 2018). Cultivars were arranged in rows of eight plots within the replicates (‘main plots’) for technical reasons. In 2014, chemical straw-shortening growth regulator (GR) was included as an additional factor (treated/non-treated). In the other years, all plots were treated with Chlormequate-based straw-shortening according to local practice. Depending on the disease incidence, adapted fungicide treatments were applied. Sowing density was 300–350 kernels m^−2^. Adequate amounts of P, K, Mg, and S were supplied.

### 2.2. Data Acquisition and Calculations

The total above-ground biomass was sampled at differing growth stages and at differing frequencies: Zadok’s growth stage (ZGS) 32 and 35 in 2009; ZGS 63 in 2010; ZGS 32 in 2011; ZGS 51 in 2013; ZGS 57 in 2014; ZGS 32 in 2016 as well as ZGS 45 and 65 in 2018 (Table 2).The sampling area per plot differed between years from 0.13 m^−2^ in 2010 to 2.7 m^−2^ in 2011. After drying at 60 °C, dry matter weight (DM [kg ha^−1^]) was determined through weighing. Nitrogen concentration (NC [%]) was determined using a ratio mass spectrometer with an ANCA SL 20-20 preparation unit (Europe Scientific, Crewe, UK) from 2009 to 2014. In the following years, near-infrared spectroscopy (NIRS) using a FOSS NIRS 6500 (NIRSystem, Silver Spring, Md.) and an FT-NIRS (Bruker, MPA, Germany) was used instead. DM and NC values were combined for the NNI as NNI = NC/(5.35 × DM^(−0.442)^) [16] and Nup [kg ha^−1^] as Nup = DM × NC.

Spectral measurements were conducted for each sampling date using the mobile sensor platform Phenotrac (I–IV). It was equipped with the same hyperspectral passive bidirectional sensor (tec5, Oberursel, Germany) [22], which measures at a nominal resolution of 3.3 nm and was used from 350 to 1000 nm. In 2018, a similarly measuring handheld hyperspectral Handyspec (tec5, Oberursel, Germany) sensor with a nominal resolution of 2.0 nm was used instead. Index comparison indicated a very close agreement between both sensors.

Spectral vegetation indices (SVI) were selected based on previous evaluation on similar traits under comparable growth conditions [22,29,30] (Table 3). The selection was limited to the REIP and simple ratio indices which were found to be more sensitive than normalized indices in dense canopies [31].

### 2.3. Data Analysis

SVIs and reference data were submitted to analysis of variance and treatment means were compared using Tukey’s HSD test. SVIs were tested in regression analysis with the target traits. The following approaches were applied using *R* version 3.4.2 [40]:

(i) Regression across the full data from different years and growth stages testing quadratic index ~ trait relationships. The minimum trait value with a non-positive slope of the index ~ trait relationship was extracted as a ’saturation point’. In addition, fitted values with negative slopes were replaced with the fitted value reached for the saturation point, resulting into quadratic + plateau curves (Figure 2) to avoid counterintuitive overfitting with declining index values for high reference trait values. The noise equivalent (NE) was calculated as root mean squared error (RMSE) divided by the 1^st^ derivative of the index values over the reference traits [28,41].

(ii) Linear or quadratic trait ~ index relationships selected based on the Akaike information criterion (AIC) within dates *across* main plot treatments (1–3 cultivars or two levels of growth regulators in 2014 only).

(iii) Linear and quadratic trait ~ index relationships selected based on the AIC within dates *within* main plot treatments.

(iv) Linear relationships within two intervals of the target traits, referring to low N status of plots with NNI < 0.8 and high N status of plots with NNI > 0.8.

Relationships were compared by coefficients of determination (R^2^), RMSE, and mean- and range-normalized RMSE.

## 3. Results

### 3.1. Treatment Effects on Target Traits and Indices

**Figure 1:**
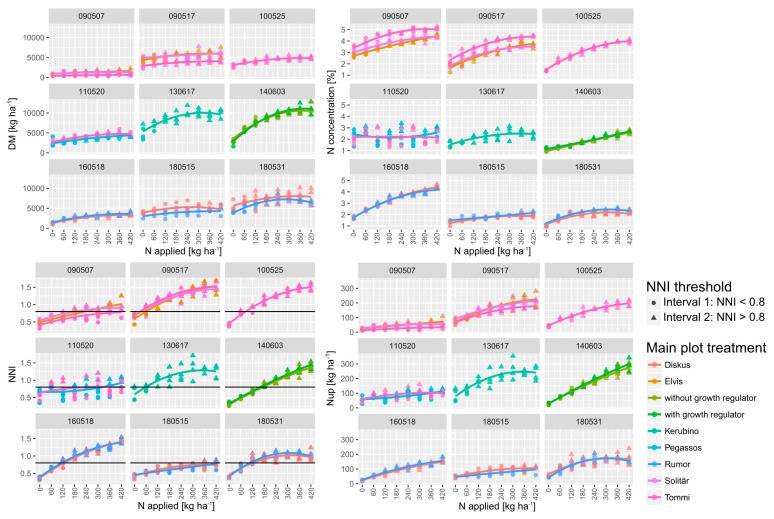
Quadratic response of reference traits to incremental N fertilization (N levels) by sampling dates (year/month/day) and main plot treatments. Nitrogen was applied incrementally increasing by 60 kg ha^−1^ from 0–420 kg N ha^−1^ in three doses (Table 1). For nitrogen nutrition index (NNI), the threshold used (NNI </> 0.8) for dividing the data into two intervals is drawn as a horizontal line. Interval 1 (NNI < 0.8) and interval 2 (NNI > 0.8) are indicated as circles and rectangles, respectively. See Supplementary Figure S1 for the N-response of the index R760_730.

**Figure 2:**
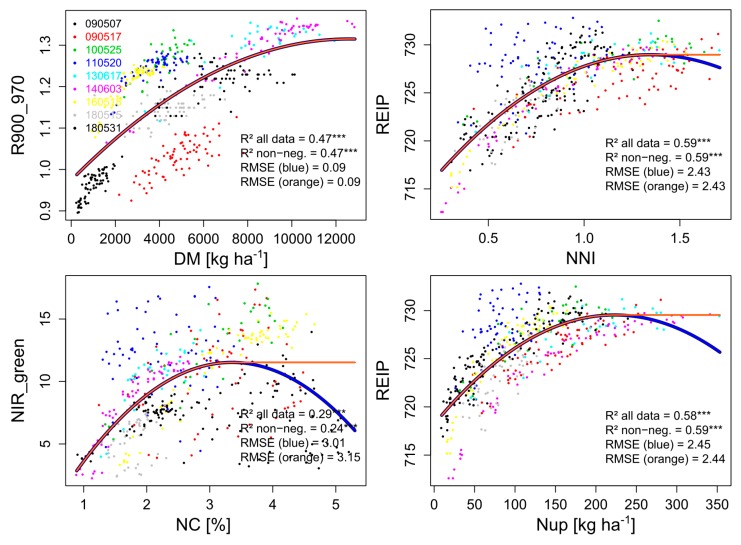
Curves fitted between reference traits and vegetation indices across all measurement dates (year/month/day) for calculating the noise equivalent (Figure 3) for the four reference traits. For each trait, the relationship (***: *p* < 0.001) of the R^2^-based best index is shown. Fitted values at negative slopes (blue line) were replaced by constant plateau-shaped values (orange line). RMSE values refer to the index values.

Owing to the different growing conditions together with the different phenology of the cultivars, growth stages differed markedly across years. In addition, the levels of reference plant traits differed substantially by growth stages by years (Figure 1; Supplementary Table S1; Supplementary Table S2). DM ranged from an average across all treatments of 1.2 t ha^−1^ at early stem elongation in 2009 to 8.4 t ha^−1^ during ear emergence in 2014. Inversely, NC ranged from the minimum 1.8% to maximum 3.9%. Nup ranged from on the average 40 kg ha^−1^ at early stem elongation 2009 (2009/05/07; dates are indicated as year/month/day) to 168 kg ha^−1^ at ear emergence in 2014 (2014/06/03).

With the exception of stem elongation in 2018 (2018/05/15), significant differences by N levels were found on all sampling dates for DM. However, the differentiation differed from 2–3 significance groups in the post hoc test in 2009, 2013, and 2018 to 5–6 groups in 2014 and 2016. For the N-related traits, more significance groups were found with the strongest effects on NNI. Among the SVIs, the N effects were the strongest on the red edge indices R760_730, R780_740, and REIP and the weakest on the R900_970 index. The vegetation indices differentiated the N levels in different ways: In general, the R780_740, the R760_730, and the REIP differentiated the best and the R900_970 the least. The level and N response of NNI differed between dates from only a few plots with an NNI > 0.8 during stem elongation in 2018 (2018/05/15) to high N status (2009/05/17 and 2010/05/25).

Analyzed across N levels, DM differed by cultivars on all dates (Supplementary Table S1), NC on all dates with the exception of stem elongation in 2016, Nup with the exception of ear emergence in 2014 and anthesis in 2018, but NNI only in 2009. The straw-shortener (growth regulator) in 2014 showed relatively low effects with no effect on DM and Nup but increased values in NC and NNI.

Both within and across measurement dates, Nup was positively related to DM (Figure 1; Supplementary Figure S2). The DM ~ NC relationships were less consistent with strong positive relationships only on the later dates in 2010, 2016 and 2014 but no significant relationships in years with stronger cultivar effects (2009 and 2011). The Nup ~ NC relationships were close within dates only. The NNI was closely related to Nup, both within and across dates, and to NC within dates and across dates with the exception of the early stem elongation date in 2009.

### 3.2. Relationships across Measurement Dates: Noise Equivalent, R^2^ and Saturation Points

The index ~ trait relationships across all dates revealed distinct differences (Table 4). For DM, only the R900_970 provided useful relationships (R^2^ = 0.47), followed by the red edge indices. In contrast, the NIR_green and NIR_red indices scattered strongly between dates (not shown). For NC, none of the indices showed a unique relationship without decreasing index values for high NC values (max. R^2^ = 0.24 of the NIR_green). The different dates were less distortive for Nup and NNI, where for both traits the REIP (R^2^ = 0.59) and both red edge simple ratio indices performed best.

**Figure 3:**
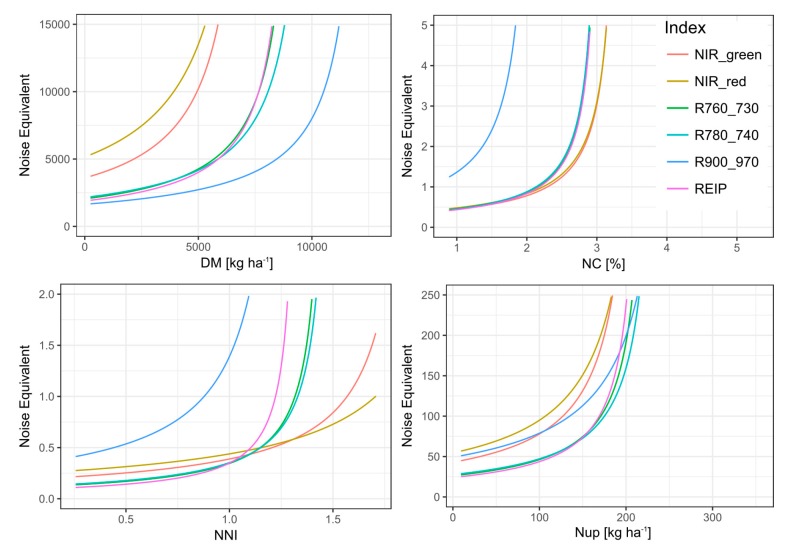
Noise equivalent for the evaluated indices (colored lines) plotted against the level of reference traits (*x*-axis) for the full data.

The range with plateau-like values was considered to be non-distinguishable by the index. Thus, it was excluded for calculating the noise equivalent (NE). Indices differed in reaching this plateau, indicating differing saturation thresholds (Supplementary Figure S4). Low saturation points were found for NC with a maximum saturation point of 3.4% (NIR_red), accompanied by steeply increasing NE values (Figure 3). In accordance with the R^2^-based ranking, only the R900_970 resolved almost the full data range (relative saturation point 0.98; 12.5 t DM ha^−1^) for DM. Correspondingly, the R900_970 showed the lowest NE values with the relatively flattest increase across the data range (Figure 3). It was followed by the similar red edge indices and higher NE values of the NIR_green and NIR_red indices. In contrast, for NNI, the index ranking differed in R^2^, NE, and saturation points. In spite of higher saturation points of the NIR_red and NIR_green indices and relatively lower NE (Figure 3) values in the upper data range, these indices showed stronger scattering in the lower data range with higher NE values and overall lower R^2^-values.

For Nup, most indices reached lower relative saturation points (0.62–0.75) than for NNI, corresponding to 223 kg N ha^−1^ (REIP, NIR_green) and 265 kg N ha^−1^ (R780_740). Clearly lowest NE values were observed for the red edge indices.

### 3.3. Index Comparisons within and across Cultivars by Measurement Dates

Within dates, regression analysis was conducted across N levels. Trait ~ index relationships were tested both across and within the main plot treatments (i.e., cultivars; growth regulator in 2014).

Generally, the influence of measurement dates was larger than the differences by main plot treatments (Supplementary Table S3, Supplementary Table S4).

For all traits, the best relationships were observed in 2014 and 2016 both within and across main plots. 2011 was characterized by non-significant relationships for NC, weak relationships for NNI (max. R^2^ = 0.15) and Nup (max. R^2^ = 0.31), but medium relationships for DM especially within the main plots. For some dates (2014/06/03, 2016/05/18, 2018/05/29), NC was equally or better (2010/05/25) estimated than NNI and Nup. Substantially weaker relationships were found for other dates (2011/05/20 and 2009/05/07).

**Figure 4:**
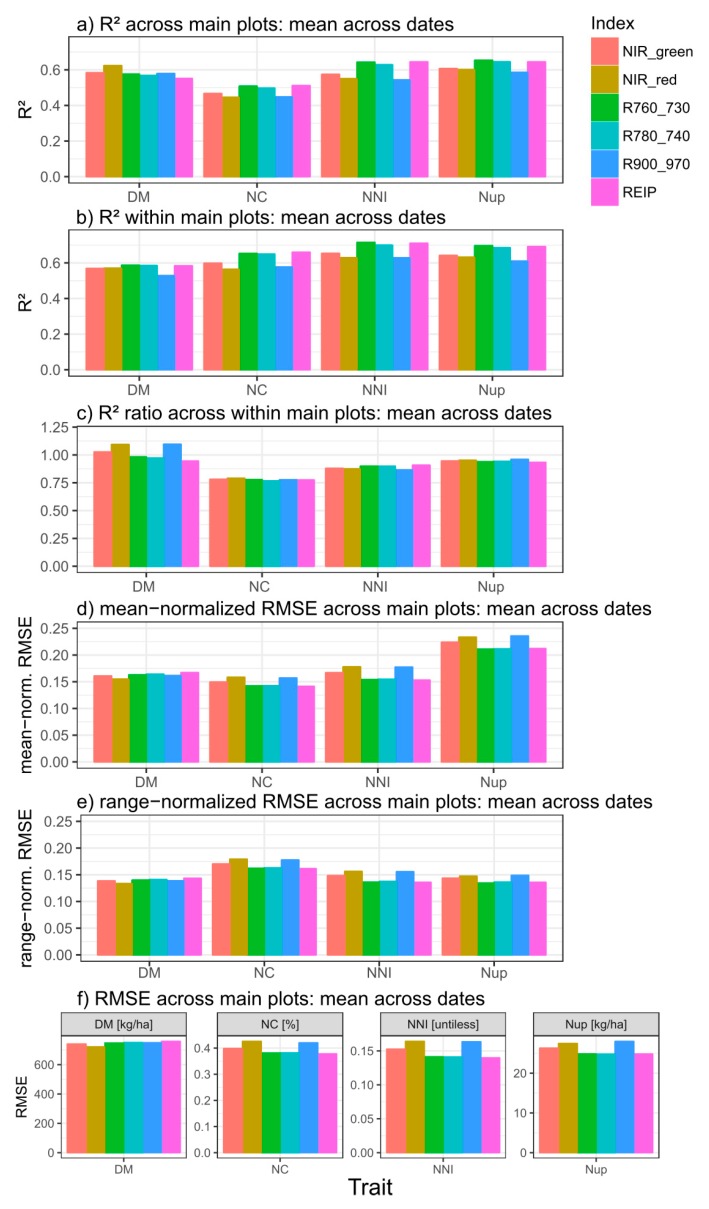
Coefficients of determination (R^2^) retrieved from linear or quadratic relationships selected based on the AIC for dry matter (DM), N concentration (NC), N nutrition index (NNI), and N uptake (Nup) averaged from the results by measurement dates, for regressions tested across (**a**; n = 9) and within (**b**; n = 18) main plot treatments. The R^2^-ratio between both approaches is visualized in (**c**). (**d**–**f**) show mean-normalized, range-normalized and absolute RMSE-values from the across-main plot approach. Non-significant relationships were included. See Supplementary Tables S3 and S4 for original results.

Compared across measurement dates, coefficients of determination (R^2^) differed overall little between the regressions across main plots and within main plots for DM. For Nup, followed by NNI and especially NC, R^2^-values were clearly lower from regressions across than within main plots (Figure 4), although all target traits differed by cultivars on most dates (Supplementary Table S1). Thus, for NC, R^2^-values decreased by approximately 25% when calculated across main plots. All vegetation indices showed a similar decrease for the N-related traits. In contrast, the NIR_red, NIR_green and R90_970 indices achieved relatively higher R^2^-values for DM across than within main plots.

For the three N-related traits, the index ranking was comparable within and across main plots. Either the REIP or the R760_730 ranked best but both indices performed very similarly and were closely followed by the R780_740 index. The other indices were clearly less suited and the explained variance was approximately 4–10% lower for the N traits. For DM, however, the NIR_red index gave on average best (R^2^ = 0.62) and the REIP weakest (R^2^ = 0.55) relationships across main plots with a similar performance of the other indices. Compared within the main plots, however, the red edge indices slightly outperformed the others for DM. Linear relationships may be preferable because they are less likely to hide saturation issues. However, for most trait*date*index combinations, the differences in R^2^ values between linear and quadratic fits were low (Supplementary Figure S3).

RMSE-values normalized to the mean and the range of the target traits generally resulted in similar index rankings (Figure 4d; Supplementary Table S5) as from the coefficients of determination (Figure 4a). However, the higher mean-normalized RMSE values for Nup than for the other traits were not reflected in lower R^2^-values. In contrast, R^2^ values were rather low for NC but mean-normalized RMSE similar as for DM and NNI.

### 3.4. Regressions by NNI-Based Intervals

**Figure 5:**
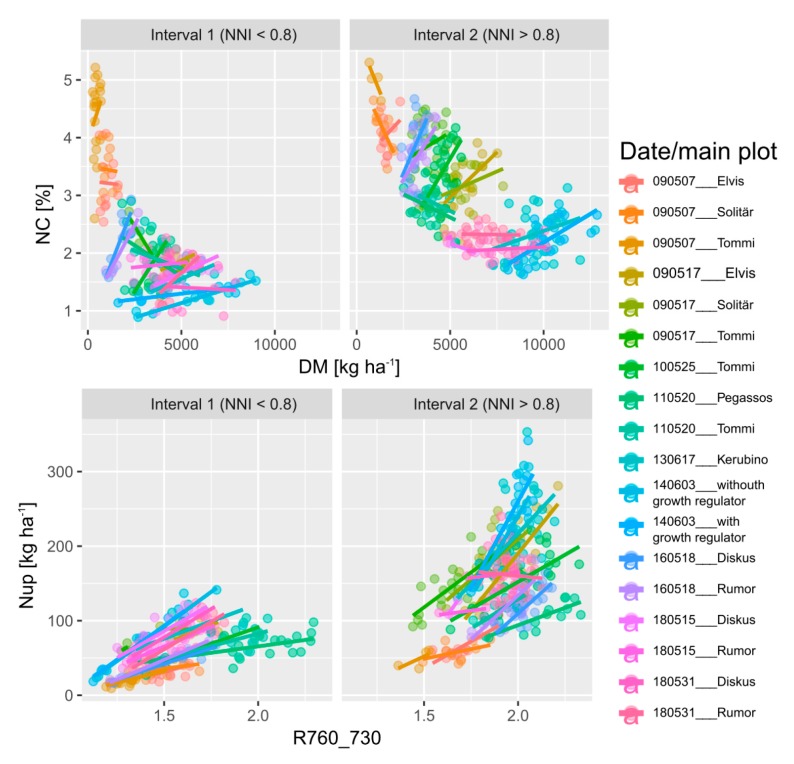
N concentration (NC) plotted against dry matter (DM) of both intervals, based on the NNI-threshold (NNI </> 0.8), and an example of the R760_730 relationships with Nup in both intervals for date*main plot combinations. Regression lines refer to linear fits within intervals. See Supplementary Figure S5 for coefficients of determination for the trait ~ index relationships.

The NNI was used for dividing the reference trait range into an interval of sufficient/excessive N supply (NNI > 0.8) and an interval with N deficiency (NNI < 0.8) for assessing the specific ability to estimate the target traits under conditions of high N status, within date*main plot combinations (Figure 5).

**Figure 6:**
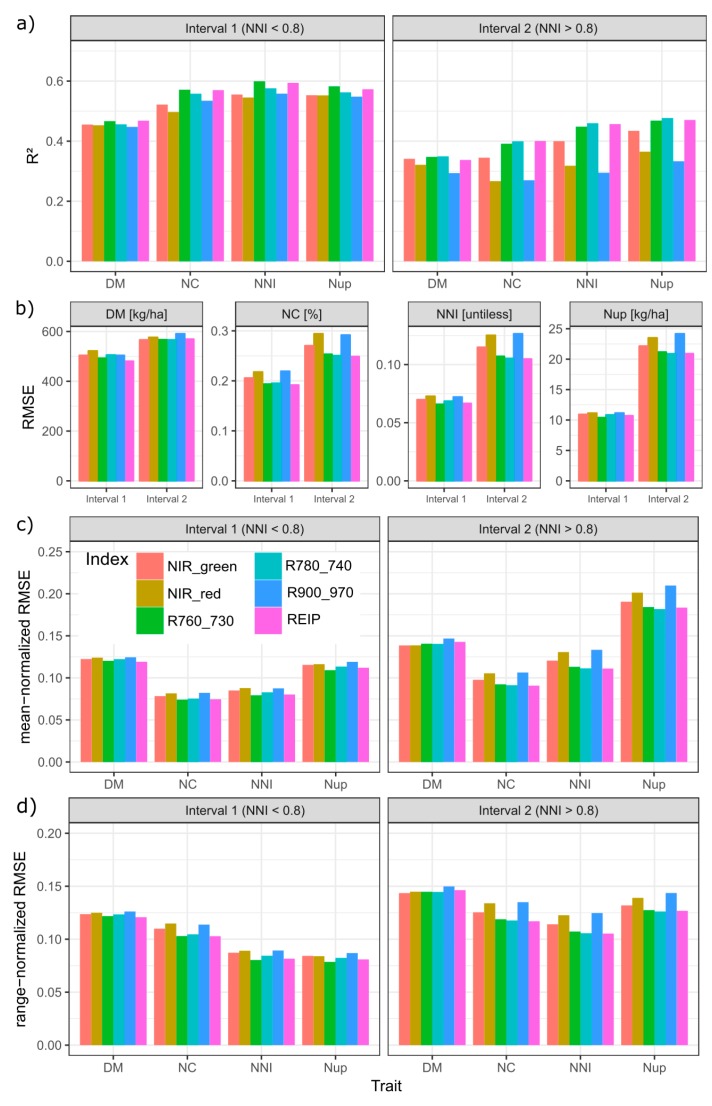
Index comparison by NNI-based intervals (interval 1: NNI < 0.8; interval 2: NNI > 0.8): (**a**) Coefficients of determination (R^2^), (**b**) absolute root mean squared error (RMSE), (**c**) mean-normalized RMSE, and (**d**) range-normalized RMSE: Averaged across results of regressions within measurement date*main plot combinations (n = 18). See Supplementary Figures S5 and S6 for original results within date*main plot combinations.

Compared by R^2^, RMSE as well as mean- and range-normalized RMSE-values, all traits were better estimated in the lower (interval 1) than in the upper (interval 2) NNI-based trait interval (Figure 6). In general, the order of the R^2^-values among traits was DM < NC ≈ NNI ≈ Nup in interval 1 and DM < NC < NNI < Nup in interval 2 (Figure 6a). Accordingly, the order by range-normalized RMSE was DM > NC > NNI ≈ Nup in interval 1 but DM > NC ≈ Nup > NNI in interval 2. However, the order by mean normalized RMSE was DM ≈ Nup > NNI ≈ NC in interval 1 and Nup > DM > NNI > NC in interval 2. For DM, all indices performed on average similarly in interval 1 (R^2^ = 0.45–0.47, RMSE = 480–520 kg ha^−1^; mean-normalized RMSE ≈ 12%) but differed more in interval 2 with the R900_970 being relatively less suited mainly due to the poor relationships during stem elongation in 2018 (Supplementary Figures S5 and S6). For all N traits, the REIP and R760_730 ranked highest (average R^2^ of the REIP = 0.40 for NNI, 0.46 for NC and 0.47 for Nup) in interval 1. In interval 2, the R780_740 reached slightly higher R^2^-values and lower absolute and normalized RMSE values than the R760_730. While the index discrimination was minor in interval 1, the R^2^, especially of the NIR_red and R900_970 in interval 2, was about 1/3 lower than that of the best-performing indices in interval 2. In spite of the overall weaker estimation in interval 2, the contrary was found for some date*main plot combinations, mainly in 2009 (Supplementary Figure S5).

## 4. Discussion

Saturation of vegetation indices is a frequently discussed limitation for the application of spectral methods in dense canopies [42,43]. This study aimed at assessing the estimation of DM, NC, NNI, and Nup under varying conditions. Dominated by the effect of N fertilization, positive trait ~ index relationships were found on all dates. An asymptotical negative NC ~ DM relationship was confirmed across dates, which was converted into the NNI [13,15].

### 4.1. The Effect of Years, Growth Stages and Cultivars

Despite the strong scattering between dates in the regression approach across dates, relevant index ~ trait relationships were found for NNI and Nup. However, unlike to Cao et al. (2018) [23] but similar to Cao et al. (2013) [24] for rice, more robust relationships across growth-stages for NNI than for Nup were not confirmed. The fact that across years, the lowest R^2^-values were found for NC in spite of mostly close relationships within dates, may indicate that these observed relationships were driven indirectly by the NC’s close relationship with DM and Nup ([25]; Supplementary Figure S2). The strong influence of growth stages for NC estimation is in line with [25]. Due to the year- and stage-specific shifts especially for DM and NC, a global model would still have resulted in substantial absolute errors. Possibly, the canopy water mass as detected by the R900_970 is a more integrative signal compared to structural NIR/VIS and NIR/red edge indices. This may explain that the latter indices were not suited across measurement dates for DM due to the concomitant influence of NC and leaf morphology, while the R900_970 was the least sensitive to NC across dates. For DM, the strongest index differentiation with lower NE of the R900_970 is in line with the previous recommendation of the water band for overcoming saturation in dense canopies [44,45]. Still, phenological shifts, which remain relevant also for the other traits and relationships across growth stages, allow only a relative assessment of the N status if growth differs too strongly [23,46,47].

For the DM and NC, a higher saturation point was associated with a higher coefficient of determination and lower NE whereas for the NNI and Nup, the differing index rankings by saturation point, NE and R^2^-values indicate that the NE alone may be misleading. The higher relative saturation points for DM and NNI than for NC and Nup should not be over-interpreted due to the strong scattering for medium trait values and the few data points contained in the upper DM and Nup range. In contrast, the low index values, which were associated with high NC values, indicate that all indices were dominantly responsive to the low DM/Nup values of these plots. The use of less structure-sensitive indices would have to be tested under such conditions [48].

The predominantly small difference between linear and quadratic relationships (Supplementary Figure S3) within growth stages for red edge indices is in line with [30]. However, it implies that the clearly curvilinear across-dates models may not be suited especially for dense canopies due to the distorting phenological effect. Moreover, the substantial different index rankings in the within-date regressions indicate that especially the across-stage robustness of the R900_970 counteracts with the performance within date*main plot conditions. This is in line with the relatively good performance of the R900_970 across main plots in contrast to within main plots for DM, possibly indicating being less sensitive to morphological differences of the cultivars. Possibly due to the more different growth stages and the different environmental conditions in the trial years, the NE level across dates and growths stages was higher than previously reported for the same target traits [46] and for Nup and NC in [28].

Testing across main plot treatments, notably cultivars, may cause ‘stretching’ of the data. However, it combines differences in morphology and phenology as well, which explains that the effect differed between traits and measurement dates. The stronger negative effect of main plots on the estimation of N traits than of DM may relate to the influence of differing chlorophyll content between cultivars [49]. The predominantly weaker relationships across than within cultivars are in line with previous findings [27,28,47]. While this aspect is relevant for discriminating genotypes in phenotyping or for the use on a landscape scale, the distortive influence is less of a problem for precision farming with commonly only one cultivar. Still, absolute errors would occur if the models were not adapted for the cultivar-specific influence.

### 4.2. The Estimation Potential Compared by Traits and Statistical Measures

Weaker index-relationships for DM and NC compared to NNI and especially Nup in all approaches (across dates, within dates, and by intervals) are in line with previous findings [22,28,29,30], and may be caused by the effect of N fertilization, which increased both DM and NC in the same ‘direction’. With both traits contributing to the Nup and NNI calculation, this may have additionally ‘stretched’ these traits. Thus, DM (but not NC) was generally less distinguishable in the post hoc test than Nup and NNI. As the coefficient of determination, the range-normalized RMSE indicated a weaker estimation of DM as well, but a similar estimation of NC and Nup in interval 2 in spite of higher R^2^-values for Nup, as well as better estimation of NNI than that of Nup regardless of a similar R^2^-level. The mean-normalized RMSE resulted in identical index rankings as the absolute and range-normalized RMSE but may be less useful to compare the trait estimability due to differing absolute levels of the traits. Despite the overall useful relationships, the estimation failed for some date*main plot combinations (Supplementary Figure S5)—mainly when the differentiation in the target traits was weak as for the very flat N response of NC in 2011 (Figure 1). Differing sampling areas and occasional temporal shifts between plant samplings and spectral measurements constitute further error sources.

The strong stage-specific clustering in the NC ~ index relationships could be addressed through combining a structural index as the NDVI with red edge information [28]. On the other hand, the NNI showed the least clustering across stages due to its immanent correction by DM, explaining its better estimation than that of NC. Thus, it may also be preferred for guiding fertilization strategies [15].

Comparing datasets and statistical measures (Supplementary Table S5), R^2^ and NE ranked the indices similarly for all traits for the whole data (‘across all’) and within date*main plots for NC, NNI, and Nup, but not for DM. The similar index rankings for NNI and Nup from all approaches did not go along with constantly weaker performance of the NIR_red and R900_970. However, due to the relatively poorer performance of the R900_970 within the main plots, the rankings within dates differed as tested across and within the main plots for DM. Still, using the whole data is not appropriate for assessing the actual index precision in the relevant data range of sufficient to excessive N supply. Besides the similar index rankings between datasets, the comparable rankings for the three N-related traits are promising for transferring algorithms between traits.

### 4.3. The Influence of the N Status

The results corroborate better trait estimation and a weaker index differentiation in sparse canopies. Although the index ranking was comparable in intervals 1 and 2, the relative advantage of the red edge indices especially over the NIR_red and R900_970 indices was clearly stronger in interval 2 and stronger for the N-related traits than for DM. The limitation of NIR_red as for the similar NDVI is well established for dense canopies [22,50,51]. The limitation of the water band information for N traits may be due to its insensitivity to NC regardless of being less prone to saturation [45]. Mostly ranking higher than the NIR_red, the NIR_green confirmed its advantage for dense canopies [32,41,52,53]. Still, red edge information appears inevitable for canopies under conditions of high-excessive N supply due to being less prone to saturation [31,41,54,55]. With the similar performance of the three red edge indices, no clear ranking could be established. The previously reported advantage of NIR/red edge simple ratio indices [22,56] and the similar CI_red-edge_ [57,58] over the REIP was not confirmed, similar as in [30]. However, a slight trade-off was found with the R760_730 ranking better than the R780_740 in interval 1 and vice versa. This may be related to a shift in the red edge position at high N status [30,58], favoring a red edge band further right. The position of the red edge band between 720 and 740 nm agrees with optimization of the related CI_red-edge_ index for chlorophyll estimation [58], and similar optimum combinations were found for in-season estimation of grain Nup and NC [44].

## 5. Conclusions

In this paper, the results obtained support the theory that spectral proximal sensing can be applied under conditions of high N status, aiming at avoiding overfertilization. While the index rankings were mostly consistent between datasets and statistical measures, the comparison of the trait estimability differed between RMSE and R^2^-values. Optimized index selection only slightly improved the estimation under conditions of low N status, indicating that simple NIR/red two-band multispectral sensors may be sufficient. In contrast, the relatively better performance of the red edge indices suggests that red edge information is crucial under conditions of high N status and in dense canopies.

## Figures and Tables

**Table 1:** Overview on field trials by years: Cultivars, dates (month/day) for three doses of N fertilization and growth regulator treatment in 2014.

Year	Cultivar	N1	N2	N3	Growth Regulator
2009	Solitär, Elvis, Tommi	04/04	05/07	05/26	
2010	Tommi	03/03	04/29	05/26	
2011	Pegassos, Tommi	03/10	04/21	05/19	
2013	Kerubino	04/08	05/08	06/17	
2014	Kerubino	02/27	04/16	05/21	1 l/ha CCC 720 (Chlormequatchlorid; Bayer, Leverkusen, D) + 0.5 l/ha Moddus (Trinexapac-ethyl; Syngenta, Basel, CH)
2016	Diskus, Rumor	03/23	04/29	05/23	
2018	Diskus, Rumor	04/10	05/07	06/05	

**Table 2:** Dates (month/day) and growth stages (Zadok’s growth stage ZGS) for reference plant sampling and spectral measurements.

Year	ZGS	Date (Plant Sampling)	Date (Spectral Measurement)	Sensor
2009	32	05/07	05/07	Phenotrac I
2009	35	05/17	05/17	Phenotrac I
2010	63	05/25	05/25	Phenotrac II
2011	32	05/10	05/20	Phenotrac III
2013	51	06/17	06/17	Phenotrac IV
2014	57	06/03	06/02	Phenotrac IV
2016	32	05/09	05/18	Phenotrac IV
2018	45	05/15	05/15	Handyspec
2018	65	05/29	05/29	Handyspec

**Table 3:** List of vegetation indices tested in this study.

Name	Equation	Reference
NIR_green	R780/R550	[32,36]
NIR_red	R780/R670	[29,37]
R760_730	R760/R730	[22]
R780_740	R780/R740	[29]
R900_970 (Water band index)	R900/R970	[38]
REIP (Red edge inflection point)	700 + 40 × ([R670 + R780]/2 – R700)/(R740 – R700)	[39]

**Table 4:** Coefficients of determination (R^2^) with significance levels (***: *p* < 0.001; n.s.: *p* > 0.05) calculated across the total data range from all dates, with non-negative slopes (Figure 2: orange curve). Values of best indices per trait are marked in bold.

Trait	NIR_green	NIR_red	R760_730	R780_740	R900_970	REIP
DM	0.07 ***	0.04 ***	0.27 ***	0.28 ***	**0.47** ***	0.29 ***
NC	**0.24** ***	0.22 ***	0.17 ***	0.16 ***	n.s.	0.19 ***
NNI	0.31 ***	0.23 ***	0.56 ***	0.56 ***	0.32 ***	**0.59** ***
Nup	0.45 ***	0.38 ***	0.55 ***	0.54 ***	0.08 ***	**0.59** ***

## Supplementary Materials

The following are available online at https://www.mdpi.com/1424-8220/19/17/3712/s1, Supplementary Table S1: Treatment effects of main plot treatment factors; Supplementary Table S2: Treatment effects of N fertilization; Supplementary Table S3: RMSE and mean-normalized RMSE for regressions across main plots; Supplementary Table S4: Significant (*p* < 0.05) coefficients of determination (R²) for the whole data and for data subsets; Supplementary Table S5: Index ranking by data and statistical approach; Supplementary Figure S1: Quadratic response of the vegetation index R760_730 to incremental N fertilization; Supplementary Figure S2: Relationships between target traits by measurement dates; Supplementary Figure S3: Coefficients of determination (R²) of the REIP within dates across main plot treatments for linear and quadratic relationships; Supplementary Figure S4: Point of saturation (plateau point) relative to the present data range and in absolute values; Supplementary Figure S5: R²-values found from linear regression analysis for both NNI-based data intervals, based on the NNI-threshold 0.8; Supplementary Figure S6: RMSE-values found from linear regression analysis for both NNI-based data intervals based on the NNI-threshold 0.8.

## Abbreviations

AIC	Akaike information criterion
CI	chlorophyll index
DM	dry matter biomass
N	Nitrogen
NC	nitrogen concentration
NE	noise equivalent
NIR	near-infrared
NNI	nitrogen nutrition index
Nup	nitrogen uptake
REIP	red edge inflection point
RMSE	root mean squared error
SVI	spectral vegetation index

## References

[1] Zhang X., Davidson E.A., Mauzerall D.L., Searchinger T.D., Dumas P., Shen Y. (2015). Managing nitrogen for sustainable development. Nature.

[2] Schlesinger W.H. (2009). On the fate of anthropogenic nitrogen. Proc. Natl. Acad. Sci. USA.

[3] Galloway J.N., Cowling E.B. (2002). Reactive nitrogen and the world: 200 years of change. AMBIO A J. Hum. Environ..

[4] Lassaletta L., Billen G., Grizzetti B., Anglade J., Garnier J. (2014). 50 year trends in nitrogen use efficiency of world cropping systems: The relationship between yield and nitrogen input to cropland. Environ. Res. Lett..

[5] Yue X.L., Hu Y., Zhang H.Z., Schmidhalter U. (2015). Green Window Approach for improving nitrogen management by farmers in small-scale wheat fields. J. Agric. Sci..

[6] Ma G., Liu W., Li S., Zhang P., Wang C. (2019). Determining the Optimal N Input to Improve Grain Yield and Quality in Winter Wheat With Reduced Apparent N Loss in the North China Plain. Front. Plant Sci..

[7] Yue X., Hu Y., Zhang H., Schmidhalter U. (2019). Optimizing the nitrogen management strategy for winter wheat in the North China Plain using rapid soil and plant nitrogen measurements. Commun. Soil Sci. Plant Anal..

[8] Zhao B., Ata-ui-karim S.T., Yao X., Tian Y., Cao W., Zhu Y., Liu X. (2016). A New Curve of Critical Nitrogen Concentration Based on Spike Dry Matter for Winter Wheat in Eastern China. PLoS ONE.

[9] Mueller N.D., Gerber J.S., Johnston M., Ray D.K., Ramankutty N., Foley J.A. (2012). Closing yield gaps through nutrient and water management. Nature.

[10] Herrera J., Rubio G., Häner L., Delgado J., Lucho-Constantino C., Islas-Valdez S., Pellet D. (2016). Emerging and established technologies to increase nitrogen use efficiency of cereals. Agronomy.

[11] Prost L., Jeuffroy M.-H. (2007). Replacing the nitrogen nutrition index by the chlorophyll meter to assess wheat N status. Agron. Sustain. Dev..

[12] Ravier C., Quemada M., Jeu M. (2017). Use of a chlorophyll meter to assess nitrogen nutrition index during the growth cycle in winter wheat Field Crops Research Use of a chlorophyll meter to assess nitrogen nutrition index during the growth cycle in winter wheat. Field Crop. Res..

[13] Gastal F., Lemaire G. (2002). N uptake and distribution in crops: An agronomical and ecophysiological perspective. J. Exp. Bot..

[14] Lemaire G., Jeuffroy M.H., Gastal F. (2008). Diagnosis tool for plant and crop N status in vegetative stage. Theory and practices for crop N management. Eur. J. Agron..

[15] Mistele B., Schmidhalter U. (2008). Estimating the nitrogen nutrition index using spectral canopy reflectance measurements. Eur. J. Agron..

[16] Justes E., Mary B., Meynard J.-M., Machet J.-M., Thelier-Huché L. (1994). Determination of a critical nitrogen dilution curve for winter wheat crops. Ann. Bot..

[17] Baret F., Houlès V., Guérif M. (2007). Quantification of plant stress using remote sensing observations and crop models: The case of nitrogen management. J. Exp. Bot..

[18] Lemaire G. (2012). Diagnosis of the Nitrogen Status in Crops.

[19] Muñoz-Huerta R.F., Guevara-Gonzalez R.G., Contreras-Medina L.M., Torres-Pacheco I., Prado-Olivarez J., Ocampo-Velazquez R.V., Muñoz-Huerta R.F., Guevara-Gonzalez R.G., Contreras-Medina L.M., Torres-Pacheco I. (2013). A review of methods for sensing the nitrogen status in plants: Advantages, disadvantages and recent advances. Sensors.

[20] Diacono M., Rubino P., Montemurro F. (2013). Precision nitrogen management of wheat. A review. Agron. Sustain. Dev..

[21] Pinter P.J., Hatfield J.L., Schepers J.S., Barnes E.M., Moran M.S., Daughtry C.S.T., Upchurch D.R. (2003). Remote sensing for crop management. Photogramm. Eng. Remote Sens..

[22] Erdle K., Mistele B., Schmidhalter U. (2011). Comparison of active and passive spectral sensors in discriminating biomass parameters and nitrogen status in wheat cultivars. Field Crop. Res..

[23] Cao Q., Miao Y., Shen J., Yuan F., Cheng S., Cui Z., Two E., Circle C., Canopy A., Status N. (2018). Evaluating Two Crop Circle Active Canopy Sensors for In-Season Diagnosis of Winter Wheat. Agronomy.

[24] Cao Q., Miao Y., Wang H., Huang S., Cheng S., Khosla R., Jiang R. (2013). Non-destructive estimation of rice plant nitrogen status with Crop Circle multispectral active canopy sensor. Field Crop. Res..

[25] Chen P. (2015). A Comparison of Two Approaches for Estimating the Wheat Nitrogen Nutrition Index Using Remote Sensing. Remote Sens..

[26] Cao Q., Miao Y., Li F., Gao X. (2017). Developing a new Crop Circle active canopy sensor-based precision nitrogen management strategy for winter. Precis. Agric..

[27] Schmid A. (2008). Erfassung des Aktuellen Stickstoffstatus von Kulturpflanzen mit Berührungsloser Sensorik zur Optimierung der Teilflächenspezifischen Bestandesführung.

[28] Li F., Mistele B., Hu Y., Yue X., Yue S., Miao Y., Chen X., Cui Z., Meng Q., Schmidhalter U. (2012). Remotely estimating aerial N status of phenologically differing winter wheat cultivars grown in contrasting climatic and geographic zones in China and Germany. Field Crop. Res..

[29] Mistele B., Gutser R., Schmidhalter U. Validation of field-scales spectral measurements of the nitrogen status in winter wheat. Proceedings of the 7th International Conference on Precision Agriculture and other Precision Resources Management.

[30] Mistele B., Schmidhalter U. (2010). Tractor-based quadrilateral spectral reflectance measurements to detect biomass and total aerial nitrogen in winter wheat. Agron. J..

[31] Nguy-Robertson A., Gitelson A., Peng Y., Viña A., Arkebauer T., Rundquist D. (2012). Green leaf area index estimation in maize and soybean: Combining vegetation indices to achieve maximal sensitivity. Agron. J..

[32] Mistele B., Schmidhalter U. (2008). Spectral measurements of the total aerial N and biomass dry weight in maize using a quadrilateral-view optic. Field Crop. Res..

[33] Li F., Zhang F., Cui Z., Li R., Chen X., Schroder J., Raun W.R. (2009). In-Season Optical Sensing Improves Nitrogen-Use Effi ciency for Winter Wheat. Soil Sci. Soc. Am. J..

[34] Jin X.L., Diao W.Y., Xiao C.H., Wang F.Y., Chen B., Wang K.R. (2015). Estimation of wheat nitrogen status under drip irrigation with canopy spectral indices. J. Agric. Sci..

[35] Li F., Mistele B., Hu Y., Chen X., Schmidhalter U. (2014). Optimising three-band spectral indices to assess aerial N concentration, N uptake and aboveground biomass of winter wheat remotely in China and Germany. ISPRS J. Photogramm. Remote Sens..

[36] Takebe M., Yoneyama T., Inada K., Murakami T. (1990). Spectral reflectance ratio of rice canopy for estimating crop nitrogen status. Plant Soil.

[37] Pearson R.L., Miller L.D. Remote mapping of standing crop biomass for estimation of the productivity of the shortgrass prairie Pawnee National Grasslands, Colorado. Proceedings of the 8th International symposium on Remote sensing of the Environment II.

[38] Peñuelas J., Filella I., Biel C., Serrano L., Savé R. (2007). The reflectance at the 950–970 nm region as an indicator of plant water status. Int. J. Remote Sens..

[39] Guyot G., Baret F., Major D.J. (1988). High spectral resolution: Determination of spectral shifts between the red and infrared. Int. Arch. Photogramm. Remote Sens..

[40] R core Team (2017). R: A Language and Environment for Statistical Computing.

[41] Viña A., Gitelson A.A. (2005). New developments in the remote estimation of the fraction of absorbed photosynthetically active radiation in crops. Geophys. Res. Lett..

[42] Cammarano D., Fitzgerald G.J., Casa R., Basso B. (2014). Assessing the Robustness of Vegetation Indices to Estimate Wheat N in Mediterranean Environments. Remote Sens..

[43] Gitelson A.A., Gritz Y., Merzlyak M.N. (2003). Relationships between leaf chlorophyll content and spectral reflectance and algorithms for non-destructive chlorophyll assessment in higher plant leaves. J. Plant Physiol..

[44] Prey L., Schmidhalter U. (2019). Simulation of satellite reflectance data using high-frequency ground based hyperspectral canopy measurements for in-season estimation of grain yield and grain nitrogen status in winter wheat. ISPRS J. Photogramm. Remote Sens..

[45] Sims D.A., Gamon J.A. (2003). Estimation of vegetation water content and photosynthetic tissue area from spectral reflectance: A comparison of indices based on liquid water and chlorophyll absorption features. Remote Sens. Environ..

[46] Cao Q., Miao Y., Feng G., Gao X., Li F., Liu B., Yue S., Cheng S., Ustin S.L., Khosla R. (2015). Active canopy sensing of winter wheat nitrogen status: An evaluation of two sensor systems. Comput. Electron. Agric..

[47] Liebler J. (2003). Feldspektroskopische Messungen zur Ermittlung des Stickstoffstatus von Winterweizen und Mais auf Heterogenen Schlägen.

[48] Blackburn G.A. (1998). Quantifying chlorophylls and caroteniods at leaf and canopy scales: An evaluation of some hyperspectral approaches. Remote Sens. Environ..

[49] Bredemeier C. (2005). Laser-Induced Chlorophyll Fluorescence Sensing as a Tool for Site-Specific Nitrogen Fertilizer Evaluation under Controlled Environmental and Field Conditions in Wheat and Maize. Ph.D. Thesis.

[50] Hatfield J.L., Prueger J.H. (2010). Value of Using Different Vegetative Indices to Quantify Agricultural Crop Characteristics at Different Growth Stages under Varying Management Practices. Remote Sens..

[51] Hatfield J.L., Gitelson A.A., Schepers J.S., Walthall C.L. (2008). Application of Spectral Remote Sensing for Agronomic Decisions. Agron. J..

[52] Gitelson A., Kaufman Y.J., Merzlyak M.N. (1996). Use of a green channel in remote sensing of global vegetation from EOS-MODIS. Remote Sens. Environ..

[53] Schmidhalter U., Jungert S., Bredemeier C., Gutser R., Manhart R., Mistele B., Gerl G., Stafford J., Werner A. (2003). Field-scale validation of a tractor based multispectral crop scanner to determine biomass and nitrogen uptake of winter wheat. Precision Agriculture, Proceedings of the 4th European Conference on Precision Agriculture, Berlin, Germany, 15–19 June 2003.

[54] Pavuluri K., Chim B.K., Griffey C.A., Reiter M.S., Balota M., Thomason W.E. (2015). Canopy spectral reflectance can predict grain nitrogen use efficiency in soft red winter wheat. Precis. Agric..

[55] Clevers J.G.P.W., Gitelson A.A. (2013). Remote estimation of crop and grass chlorophyll and nitrogen content using red-edge bands on sentinel-2 and-3. Int. J. Appl. Earth Obs. Geoinf..

[56] Heege H.J., Reusch S., Thiessen E. (2008). Prospects and results for optical systems for site-specific on-the-go control of nitrogen-top-dressing in Germany. Precis. Agric..

[57] Clevers J.G.P.W., Kooistra L. (2012). Using Hyperspectral Remote Sensing Data for Retrieving Canopy Chlorophyll and Nitrogen Content. IEEE J. Sel. Top. Appl. Earth Obs. Remote Sens..

[58] Schlemmer M., Schepers J.S., Ferguson R.B. (2013). Remote estimation of nitrogen and chlorophyll contents in maize at leaf and canopy levels. Int. J. Appl. Earth Obs. Geoinf..

