# Supplementary material for: Sensitivity of Vegetation Indices for Estimating Vegetative N Status in Winter Wheat
Source: Sensors (Basel). 2019 Aug 27;19(17):3712. doi: 10.3390/s19173712 (PMC6749256; doi:10.3390/s19173712)
Supplement: Supplementary file 1 [file sensors-19-03712-s001.pdf]

Supplementary Materials

Supplementary Table 1: Treatment effects of main plot treatment factors (Cultivars; growth regulator in 2014) on the reference traits dry matter (DM), N concentration (NC), and N uptake (Nup) and tested/selected vegetation indices for years with differing main plot factors.

date	main plot	DM [100 kg ha^{-1}]		NC [%]		Nup [kg ha^{-1}]		NNI		NIR_green		NIR_red		R760_730		R780_740		R900_970		REIP	
090507	Solitär	12	a	3.9	b	47	a	0.77	a	8.7	b	19.5	b	1.6	a	1.28	a	0.97	b	724	a
090507	Elvis	13	a	3.6	c	49	a	0.76	a	9.5	a	24.5	a	1.6	a	1.29	a	0.99	a	724	a
090507	Tommi	5	b	4.5	a	25	b	0.64	b	4.7	c	6.6	c	1.3	b	1.18	b	0.93	c	720	b
090517	Solitär	55	a	2.9	b	164	a	1.17	a	10.8	b	27.2	b	1.8	b	1.39	b	1.03	b	727	b
090517	Elvis	55	a	2.9	b	162	a	1.16	a	13.1	a	34.1	a	1.9	a	1.43	a	1.07	a	728	a
090517	Tommi	37	b	3.7	a	136	b	1.22	a	7.7	c	12.6	c	1.6	c	1.30	c	0.99	c	725	c
110510	Pegassos	36	b	2.3	a	81	a	0.74	a	13.0	a	25.7	b	2.0	a	1.50	a	1.24	a	729	a
110510	Tommi	41	a	2.2	a	89	a	0.76	a	11.5	b	33.5	a	1.9	b	1.42	b	1.25	a	727	b
140603	GR_0	84	a	1.8	b	164	a	0.89	b	8.6	a	21.8	a	1.8	a	1.36	a	1.29	a	725	a
140603	GR_1	84	a	1.9	a	173	a	0.93	a	8.5	b	20.3	b	1.7	b	1.35	a	1.28	b	725	a
160509	Diskus	27	b	3.3	a	94	b	0.97	a	11.6	a	30.4	b	1.9	a	1.39	a	1.20	b	726	a
160509	Rumor	29	a	3.3	a	102	a	1	a	11.4	a	38.9	a	1.8	b	1.37	b	1.22	a	725	b
180515	Diskus	48	a	1.7	b	86	a	0.65	a	5.2	a	10.3	a	1.5	b	1.22	b	1.15	a	722	b
180515	Rumor	39	b	1.9	a	75	b	0.64	a	5.4	a	10.0	a	1.5	a	1.24	a	1.13	b	723	a
180529	Diskus	73	a	1.9	b	140	a	0.85	a	7.2	a	11.5	a	1.8	b	1.40	b	1.21	b	727	b
180529	Rumor	62	b	2.1	a	134	a	0.88	a	7.0	a	11.0	b	1.9	a	1.43	a	1.23	a	728	a

Supplementary Table 2: Treatment effects of N fertilization (N level) on the reference traits dry matter (DM), N concentration (NC) and N uptake (Nup) and tested/selected vegetation indices.

Date (year/ month/ day)	N level	DM [100 kg ha^{-1}]		NC [%]		Nup [kg ha^{-1}]		NNI		NIR_red		NIR_green		R760_730		R780_740		R900_970		REIP	
090507	1	7	b	3.0	f	20	d	0.5	d	11.5	c	5.7	e	1.4	e	1.18	d	0.95	d	720	d
090507	2	10	ab	3.4	e	31	cd	0.6	cd	13.5	bc	6.4	de	1.4	de	1.21	cd	0.95	cd	721	cd
090507	3	10	ab	3.8	d	34	cd	0.7	bc	15.7	abc	7.1	cde	1.5	cd	1.23	bc	0.96	bcd	722	bc
090507	4	11	a	4.1	c	44	abc	0.8	ab	16.2	abc	7.5	bcd	1.5	bcd	1.25	bc	0.96	abcd	723	b
090507	5	11	a	4.1	bc	45	abc	0.8	ab	18.3	ab	8.1	abc	1.6	abc	1.27	ab	0.97	abc	723	ab
090507	6	9	ab	4.4	ab	39	bc	0.8	b	17.3	abc	8.0	abcd	1.6	abc	1.27	ab	0.97	abc	723	ab
090507	7	13	a	4.4	ab	57	a	0.9	a	21.7	a	9.3	a	1.6	a	1.31	a	0.98	a	724	a
090517	8	12	a	4.7	a	54	ab	0.9	a	20.7	a	9.0	ab	1.6	ab	1.30	a	0.97	ab	724	a
090517	1	41	c	1.9	f	76	d	0.7	f	12.8	e	6.6	e	1.5	e	1.23	e	0.99	d	722	e
090517	2	43	bc	2.4	e	101	d	0.8	e	18.5	de	8.7	d	1.6	d	1.31	d	1.01	c	725	d
090517	3	49	abc	2.9	d	138	c	1.1	d	23.0	cd	10.0	cd	1.7	cd	1.36	cd	1.03	bc	726	c
090517	4	52	ab	3.2	cd	163	bc	1.2	c	24.7	bcd	10.7	bc	1.8	bc	1.38	bc	1.03	abc	727	bc
090517	5	47	abc	3.4	bc	159	bc	1.3	bc	27.5	abc	11.3	abc	1.9	abc	1.41	abc	1.04	ab	727	ab
090517	6	53	a	3.7	ab	188	ab	1.4	ab	27.1	abc	11.5	abc	1.9	ab	1.41	ab	1.04	ab	727	ab
090517	7	54	a	3.9	a	205	a	1.5	a	32.5	a	12.8	a	1.9	a	1.45	a	1.05	a	728	a
090517	8	53	a	3.9	a	203	a	1.5	a	31.0	ab	12.4	ab	1.9	a	1.44	a	1.05	a	728	a
100525	1	29	d	1.4	f	41	e	0.4	e	10.4	d	6.0	d	1.5	d	1.23	d	1.19	d	722	d
100525	2	40	bc	2.3	e	89	d	0.8	d	20.9	cd	9.8	c	1.8	c	1.36	c	1.24	c	726	c
100525	3	39	c	2.6	d	102	d	0.9	d	25.9	bc	11.4	bc	1.9	bc	1.41	bc	1.26	bc	727	bc
100525	4	45	abc	3.1	c	137	c	1.1	c	33.8	abc	13.4	ab	2.0	ab	1.47	ab	1.27	abc	729	ab
100525	5	47	abc	3.4	bc	159	bc	1.3	bc	37.2	ab	14.5	ab	2.1	a	1.50	ab	1.29	ab	729	ab
100525	6	47	ab	3.7	ab	172	ab	1.4	ab	40.4	ab	15.3	a	2.1	a	1.53	a	1.29	ab	730	a
100525	7	49	a	4.0	a	192	a	1.5	a	39.4	ab	15.1	a	2.1	a	1.52	a	1.30	a	730	a
100525	8	49	a	4.0	a	196	a	1.5	a	42.6	a	15.9	a	2.2	a	1.55	a	1.31	a	731	a
110510	1	26	d	2.1	ab	52	c	0.6	c	14.1	d	6.7	d	1.5	c	1.25	d	1.19	c	723	d
110510	2	30	cd	2.7	a	81	abc	0.8	ab	24.4	c	10.3	c	1.8	b	1.39	c	1.24	b	727	c
110510	3	36	bc	2.1	ab	74	bc	0.7	abc	28.1	bc	11.5	bc	1.9	b	1.43	c	1.24	ab	728	c
110510	4	37	bc	2.0	b	73	bc	0.7	bc	32.4	ab	13.1	ab	2.0	a	1.49	b	1.26	ab	729	b
110510	5	42	ab	2.4	ab	99	ab	0.8	ab	32.8	ab	13.4	a	2.1	a	1.50	ab	1.26	ab	730	ab
110510	6	43	ab	2.2	ab	98	ab	0.8	abc	35.0	a	14.3	a	2.1	a	1.53	ab	1.26	a	730	ab
110510	7	47	a	2.0	b	93	ab	0.7	abc	33.6	ab	13.9	a	2.1	a	1.52	ab	1.26	a	730	ab
110510	8	44	ab	2.6	ab	111	a	0.9	a	36.1	a	14.7	a	2.1	a	1.55	a	1.26	a	730	a
130617	1	52	c	1.6	c	86	d	0.6	d	11.6	b	6.1	b	1.5	b	1.26	c	1.19	b	723	c
130617	2	70	bc	1.7	bc	122	cd	0.8	cd	22.8	a	9.3	ab	1.8	a	1.39	bc	1.28	a	726	b
130617	3	85	ab	2.1	abc	180	bc	1	bc	25.1	a	9.9	a	1.9	a	1.43	ab	1.30	a	727	ab
130617	4	87	ab	2.2	abc	194	b	1.1	abc	29.0	a	11.2	a	2.0	a	1.48	ab	1.32	a	729	ab
130617	5	102	a	2.3	ab	228	ab	1.2	ab	31.0	a	11.8	a	2.1	a	1.50	ab	1.33	a	729	ab
130617	6	101	a	2.6	a	264	a	1.4	a	29.0	a	11.1	a	2.0	a	1.49	ab	1.32	a	729	ab
130617	7	92	ab	2.6	a	241	ab	1.3	ab	32.8	a	12.4	a	2.1	a	1.53	a	1.33	a	730	a
130617	8	100	a	2.3	ab	236	ab	1.2	ab	30.5	a	11.6	a	2.0	a	1.50	ab	1.33	a	729	ab
140603	1	27	f	1.1	e	29	f	0.3	h	2.9	g	2.7	g	1.2	g	1.11	g	1.10	f	714	g
140603	2	59	e	1.3	de	74	e	0.5	g	8.6	f	5.0	f	1.4	f	1.19	f	1.21	e	720	f
140603	3	77	d	1.4	d	109	d	0.7	f	16.5	e	7.3	e	1.6	e	1.27	e	1.27	d	723	e
140603	4	87	cd	1.8	c	152	c	0.9	e	23.1	d	9.1	d	1.8	d	1.35	d	1.31	c	726	d
140603	5	95	bc	1.9	c	183	c	1	d	26.6	c	10.1	c	1.9	c	1.42	c	1.33	b	727	c
140603	6	103	ab	2.2	b	227	b	1.2	c	28.3	b	10.6	b	2.0	b	1.46	b	1.34	ab	728	b

140603	7	107	a	2.4	b	256	ab	1.3	b	29.7	ab	11.2	a	2.0	a	1.49	a	1.35	a	729	a
140603	8	110	a	2.6	a	287	a	1.4	a	30.0	a	11.3	a	2.0	a	1.50	a	1.35	a	729	a
160509	1	12	e	1.7	g	21	g	0.4	g	6.9	d	4.1	e	1.3	f	1.14	f	1.10	d	717	f
160509	2	22	d	2.4	f	51	f	0.6	f	21.6	c	7.9	d	1.6	e	1.25	e	1.18	c	722	e
160509	3	26	cd	2.9	e	77	e	0.8	e	36.1	b	11.1	c	1.8	d	1.34	d	1.21	b	725	d
160509	4	30	bc	3.3	d	98	d	1	d	42.3	a	12.9	b	1.9	c	1.41	c	1.23	a	727	c
160509	5	30	bc	3.7	c	110	cd	1.1	c	43.6	a	13.7	ab	2.0	b	1.45	b	1.24	a	728	bc
160509	6	33	ab	3.8	bc	127	bc	1.2	bc	43.5	a	14.0	ab	2.0	ab	1.47	b	1.24	a	729	ab
160509	7	33	ab	4.0	b	131	b	1.3	b	41.8	a	14.0	ab	2.1	ab	1.48	ab	1.23	a	729	ab
160509	8	36	a	4.4	a	159	a	1.5	a	42.0	a	14.3	a	2.1	a	1.51	a	1.24	a	730	a
180515	1	34	a	1.3	d	47	d	0.4	d	6.3	d	3.8	d	1.3	f	1.15	f	1.11	c	720	f
180515	2	40	a	1.6	cd	62	cd	0.5	cd	7.6	cd	4.3	cd	1.4	ef	1.17	ef	1.12	c	721	ef
180515	3	42	a	1.7	bc	68	bcd	0.6	bcd	9.0	bc	4.9	bc	1.4	de	1.20	de	1.14	bc	722	de
180515	4	45	a	1.7	abc	80	abc	0.6	abc	10.6	ab	5.5	ab	1.5	cd	1.23	cd	1.15	ab	723	cd
180515	5	46	a	1.8	ab	85	abc	0.7	abc	11.1	ab	5.6	ab	1.6	bc	1.25	bc	1.15	ab	723	bc
180515	6	48	a	1.9	ab	94	abc	0.7	ab	11.3	ab	5.8	a	1.6	abc	1.26	abc	1.15	ab	724	abc
180515	7	48	a	2.0	a	95	ab	0.7	a	12.6	a	6.3	a	1.6	ab	1.29	ab	1.16	a	725	ab
180515	8	44	a	2.0	a	101	a	0.8	a	12.6	a	6.3	a	1.7	a	1.29	a	1.16	a	725	a
180529	1	47	b	1.1	c	49	c	0.4	d	5.4	d	4.1	d	1.4	e	1.19	e	1.14	e	721	e
180529	2	59	ab	1.7	b	99	b	0.7	c	9.2	c	6.1	c	1.7	d	1.33	d	1.19	d	726	d
180529	3	65	ab	2.1	a	135	ab	0.9	b	11.0	b	6.9	b	1.8	c	1.39	c	1.21	c	728	c
180529	4	70	a	2.1	a	148	a	0.9	ab	12.6	a	7.8	a	1.9	b	1.46	b	1.23	b	729	b
180529	5	75	a	2.2	a	162	a	1	ab	12.7	a	7.9	a	1.9	ab	1.47	ab	1.24	ab	730	ab
180529	6	78	a	2.2	a	169	a	1	ab	12.7	a	7.9	a	2.0	ab	1.48	ab	1.24	ab	730	ab
180529	7	76	a	2.3	a	173	a	1	a	13.0	a	8.0	a	2.0	ab	1.49	a	1.24	ab	730	a
180529	8	71	a	2.3	a	161	a	1	ab	13.4	a	8.2	a	2.0	a	1.51	a	1.25	a	730	a

Supplementary Table 3: RMSE and mean-normalized RMSE for regressions across main plots (Cultivars; growth regulator in 2014).

		RMSE							mean-normalized RMSE					
date	trait	NIR_green	NIR_red	R760_730	R780_740	R900_970	REIP	trait level	NIR_green	NIR_red	R760_730	R780_740	R900_970	REIP
090507	DM [kg ha-1]	260	258	269	276	266	284	10	25%	25%	26%	27%	26%	28%
090517		780	704	778	783	761	821	49	16%	14%	16%	16%	16%	17%
100525		524	535	519	528	487	530	43	12%	12%	12%	12%	11%	12%
110510		626	487	641	656	610	675	38	16%	13%	17%	17%	16%	18%
130617		1255	1257	1239	1220	1336	1230	86	15%	15%	14%	14%	16%	14%
140603		738	827	729	746	727	696	84	9%	10%	9%	9%	9%	8%
160509		342	350	341	334	322	348	28	12%	13%	12%	12%	12%	12%
180515		911	841	913	930	956	933	43	21%	19%	21%	21%	22%	21%
180529		1233	1238	1297	1279	1285	1314	68	18%	18%	19%	19%	19%	19%
090507	NC [%]	0.67	0.65	0.69	0.69	0.67	0.69	4.0	17%	16%	17%	17%	17%	17%
090517		0.76	0.78	0.73	0.72	0.78	0.70	3.2	24%	25%	23%	23%	25%	22%
100525		0.42	0.44	0.39	0.41	0.42	0.39	3.0	14%	15%	13%	13%	14%	13%
110510		0.58	0.58	0.58	0.58	0.58	0.58	2.3	26%	26%	26%	26%	26%	26%
130617		0.31	0.31	0.31	0.31	0.35	0.31	2.2	14%	14%	14%	14%	16%	14%
140603		0.16	0.18	0.15	0.15	0.21	0.16	1.9	9%	10%	8%	8%	11%	8%
160509		0.26	0.46	0.21	0.19	0.38	0.20	3.3	8%	14%	6%	6%	11%	6%
180515		0.24	0.23	0.20	0.22	0.22	0.21	1.8	13%	13%	11%	13%	12%	12%
180529		0.20	0.20	0.17	0.17	0.18	0.17	1.98	10%	10%	9%	8%	9%	8%
090507	NNI	0.13	0.14	0.12	0.11	0.14	0.11	0.7	18%	19%	16%	16%	20%	15%
090517		0.26	0.27	0.23	0.23	0.28	0.21	1.2	22%	23%	20%	19%	24%	18%
100525		0.19	0.20	0.18	0.19	0.18	0.19	1.1	17%	18%	16%	17%	17%	17%
110510		0.19	0.19	0.19	0.19	0.19	0.19	0.8	26%	26%	26%	26%	26%	26%
130617		0.19	0.19	0.19	0.18	0.21	0.18	1.1	18%	18%	17%	17%	19%	17%
140603		0.10	0.11	0.09	0.09	0.13	0.09	0.9	11%	13%	10%	10%	14%	10%
160509		0.11	0.18	0.10	0.09	0.14	0.10	1.0	11%	18%	10%	9%	15%	10%
180515		0.10	0.09	0.08	0.09	0.09	0.09	0.6	16%	14%	12%	15%	14%	14%
180529		0.10	0.10	0.10	0.09	0.11	0.10	0.87	12%	12%	11%	11%	12%	11%
090507	Nup [kg ha-1]	11	12	10	10	12	10	40	27%	29%	26%	25%	30%	25%
090517		36	38	31	30	41	28	154	23%	25%	20%	19%	26%	18%
100525		32	32	30	32	29	31	136	23%	24%	22%	23%	21%	23%
110510		24	23	24	24	24	25	85	28%	27%	28%	28%	28%	29%
130617		46	46	45	44	50	45	194	24%	24%	23%	23%	26%	23%
140603		25	29	24	22	31	23	168	15%	17%	14%	13%	19%	14%
160509		18	24	16	15	20	16	98	18%	25%	17%	16%	20%	16%
180515		19	16	16	18	17	17	80	24%	20%	20%	22%	21%	21%
180529		27	27	27	27	29	28	137	20%	20%	20%	20%	21%	20%

Supplementary Table 4: Significant ($p < 0.05$) coefficients of determination (R^2) for the whole data and for data subsets (by main plots: Cultivars; growth regulator in 2014 as indicated in **Error! Reference source not found.**), calculated across N levels.

		across main plots						Main plot 1						Main plot 2						Main plot 3					
trait	date	NIR_green	NIR_red	R760_730	R780_740	R900_970	REIP	NIR_green	NIR_red	R760_730	R780_740	R900_970	REIP	NIR_green	NIR_red	R760_730	R780_740	R900_970	REIP	NIR_green	NIR_red	R760_730	R780_740	R900_970	REIP
DM	090507	0.71	0.71	0.69	0.67	0.7	0.65	0.3	0.36	0.28	0.28	0.35	0.27	0.55	0.55	0.54	0.53	0.57	0.55	0.56	0.6	0.57	0.58	0.47	0.54
DM	090517	0.54	0.63	0.55	0.54	0.57	0.49							0.48	0.49	0.54	0.55	0.6	0.53	0.48	0.44	0.49	0.5	0.44	0.49
DM	100525	0.53	0.51	0.54	0.52	0.59	0.52	0.53	0.51	0.54	0.52	0.59	0.52												
DM	110510	0.5	0.7	0.47	0.45	0.52	0.42	0.75	0.79	0.73	0.72	0.74	0.73	0.69	0.59	0.7	0.7	0.48	0.71						
DM	130617	0.58	0.58	0.59	0.6	0.52	0.59	0.58	0.58	0.59	0.6	0.52	0.59												
DM	140603	0.92	0.9	0.93	0.92	0.93	0.93	0.94	0.93	0.94	0.94	0.94	0.95	0.91	0.89	0.91	0.91	0.92	0.92						
DM	160509	0.81	0.81	0.82	0.82	0.84	0.81	0.87	0.84	0.88	0.88	0.87	0.88	0.83	0.77	0.85	0.85	0.8	0.85						
DM	180515	0.3	0.41	0.3	0.28	0.23	0.27	0.36	0.56	0.55	0.48	0.24	0.51	0.47	0.46	0.48	0.5		0.52						
DM	180529	0.36	0.36	0.29	0.31	0.31	0.28	0.52	0.52	0.53	0.54	0.53	0.5	0.32	0.29	0.31	0.31	0.32	0.31						
NC	090507		0.11					0.54	0.45	0.62	0.63	0.38	0.63	0.49	0.29	0.6	0.61	0.32	0.64	0.44	0.34	0.59	0.65	0.29	0.67
NC	090517			0.12	0.14		0.2	0.76	0.77	0.8	0.82	0.53	0.82	0.72	0.67	0.77	0.77	0.67	0.78	0.7	0.58	0.75	0.76	0.68	0.78
NC	100525	0.76	0.73	0.8	0.77	0.75	0.79	0.76	0.73	0.8	0.77	0.75	0.79												
NC	110510																								
NC	130617	0.55	0.54	0.54	0.57	0.45	0.55	0.55	0.54	0.54	0.57	0.45	0.55												
NC	140603	0.91	0.88	0.92	0.92	0.84	0.91	0.95	0.95	0.95	0.96	0.89	0.95	0.89	0.87	0.9	0.9	0.81	0.9						
NC	160509	0.91	0.71	0.94	0.95	0.81	0.95	0.91	0.82	0.95	0.96	0.87	0.96	0.92	0.77	0.95	0.96	0.86	0.96						
NC	180515	0.17	0.25	0.41	0.27	0.31	0.34			0.32		0.75	0.29	0.2	0.41	0.46	0.33	0.46	0.4						
NC	180529	0.79	0.78	0.84	0.85	0.82	0.85	0.9	0.89	0.92	0.92	0.86	0.92	0.82	0.82	0.82	0.83	0.8	0.82						
NNI	090507	0.5	0.41	0.59	0.62	0.39	0.63	0.59	0.5	0.64	0.64	0.41	0.66	0.69	0.54	0.75	0.76	0.58	0.76	0.7	0.64	0.75	0.77	0.6	0.76
NNI	090517	0.29	0.2	0.43	0.45	0.15	0.51	0.66	0.7	0.74	0.75	0.46	0.76	0.75	0.66	0.81	0.81	0.75	0.81	0.79	0.7	0.84	0.85	0.76	0.86
NNI	100525	0.72	0.7	0.75	0.72	0.74	0.74	0.72	0.7	0.75	0.72	0.74	0.74												
NNI	110510	0.11	0.1	0.11	0.1	0.09	0.1	0.15	0.12	0.14	0.14		0.14	0.12		0.12									

NNI	130617	0.6	0.6	0.61	0.62	0.51	0.62	0.6	0.6	0.61	0.62	0.51	0.62												
NNI	140603	0.93	0.9	0.94	0.94	0.88	0.94	0.96	0.95	0.97	0.97	0.92	0.97	0.9	0.89	0.91	0.92	0.84	0.92						
NNI	160509	0.89	0.74	0.92	0.93	0.83	0.93	0.91	0.83	0.95	0.96	0.87	0.96	0.9	0.76	0.94	0.95	0.84	0.95						
NNI	180515	0.33	0.51	0.61	0.44	0.52	0.52	0.26	0.43	0.62	0.43	0.76	0.51	0.43	0.58	0.67	0.52	0.47	0.61						
NNI	180529	0.8	0.79	0.82	0.83	0.78	0.81	0.84	0.83	0.86	0.86	0.81	0.85	0.79	0.78	0.79	0.8	0.78	0.78						
Nup	090507	0.69	0.63	0.72	0.73	0.62	0.72	0.48	0.5	0.49	0.49	0.4	0.49	0.73	0.66	0.76	0.76	0.68	0.75	0.66	0.67	0.66	0.67	0.56	0.66
Nup	090517	0.51	0.45	0.64	0.66	0.37	0.69	0.52	0.54	0.61	0.62	0.36	0.62	0.73	0.67	0.79	0.8	0.77	0.82	0.78	0.71	0.8	0.83	0.71	0.84
Nup	100525	0.65	0.64	0.68	0.65	0.71	0.66	0.65	0.64	0.68	0.65	0.71	0.66												
Nup	110510	0.26	0.32	0.25	0.25	0.26	0.22	0.41	0.38	0.39	0.38	0.33	0.37	0.32	0.28	0.32	0.32	0.25	0.32						
Nup	130617	0.56	0.57	0.58	0.6	0.49	0.59	0.56	0.57	0.58	0.6	0.49	0.59												
Nup	140603	0.92	0.89	0.92	0.93	0.87	0.93	0.94	0.93	0.95	0.96	0.91	0.96	0.9	0.88	0.9	0.91	0.84	0.9						
Nup	160509	0.84	0.7	0.86	0.88	0.8	0.87	0.88	0.79	0.92	0.93	0.84	0.93	0.85	0.69	0.9	0.91	0.78	0.91						
Nup	180515	0.36	0.55	0.56	0.43	0.52	0.48	0.27	0.51	0.68	0.51	0.63	0.58	0.53	0.64	0.74	0.6	0.39	0.68						
Nup	180529	0.67	0.66	0.67	0.67	0.63	0.64	0.72	0.71	0.73	0.74	0.7	0.72	0.62	0.6	0.63	0.64	0.63	0.64						

Supplementary Table 5: Index ranking by data and statistical approach. Absolute RMSE (not shown) and normalized RMSE values result in same index rankings. Two numbers indicate differing rankings depending on the data level.

Data	approach	NIR_green	NIR_red	R760_730	R780_740	R900_970	REIP	NIR_green	NIR_red	R760_730	R780_740	R900_970	REIP	NIR_green	NIR_red	R760_730	R780_740	R900_970	REIP	NIR_green	NIR_red	R760_730	R780_740	R900_970	REIP
		DM						NC						NNI						Nup					
across all	NE (lower/ upper range)	5	4	3	2	1	3	1	1	2	2	3	2	4/2	5/1	2/3	2/3	6	1/5	4	5	1	1	3	1
across all	R^2	3	3	2	2	1	2	1	1	2	2	3	2	3	4	2	2	3	1	4	5	2	3	6	1
within dates across MPs	R^2	2	1	2	2	2	3	3	4	1	2	4	1	3	4	1	2	4	1	3	3	1	2	3	1
within dates across MPs	mean-norm. RMSE	2	1	2	2	2	3	2	4	1	1	4	1	2	3	1	1	3	1	2	3	1	1	3	1
within date*MPs	R^2	2	2	1	1	2	1	4	5	2	3	5	1	3	4	1	2	4	1	3	4	1	2	5	1
within dates*MPs	NE	1	1	1	1	2	1	5	6	3	2	4	1	4	6	1	3	5	1	4	6	2	3	5	1
within date*MPs	y-interval 1: R^2	3	3	1	2	3	1	4	5	1	2	3	1	4	5	1	3	4	1	4	4	1	3	4	2
within date*MPs	y-interval 2: R^2	2	3	1	1	3	2	3	4	2	1	4	1	4	5	3	2	6	1	3	4	2	1	5	2
within date*MPs	y-interval 1: mean-norm. RMSE	2	3	1	2	3	1	3	4	1	2	4	1	3	4	1	2	4	1	4	5	1	3	6	2
within date*MPs	y-interval 2: mean-norm. RMSE	1	2	2	2	4	3	3	4	2	1	4	1	3	4	2	1	5	1	3	4	2	1	5	1

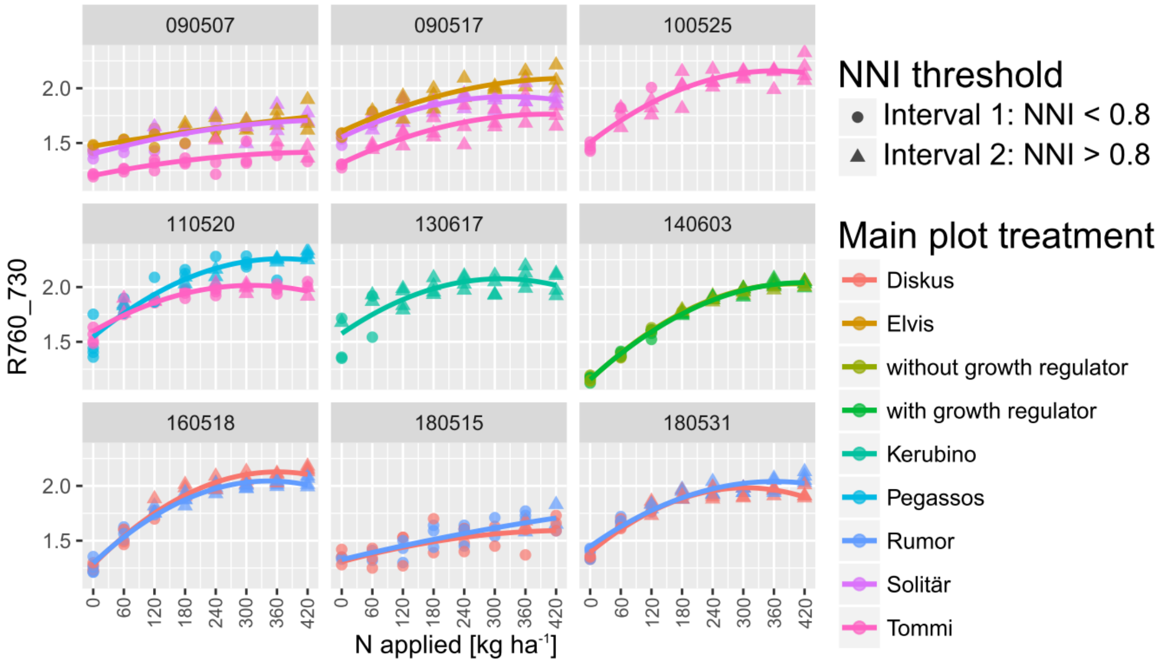

Supplementary Figure 1: Quadratic response of the vegetation index R760_730 to incremental N fertilization (N levels) by sampling dates (year/month/day) and main plot treatments. The applied nitrogen corresponds to 0–420 kg N ha^{-1} in total fertilized nitrogen, applied in four doses (**Error! Reference source not found.**). For NNI, the threshold used (NNI </> 0.8) for dividing the data into two intervals is drawn as a horizontal line. Interval 1 (NNI < 0.8) and interval 2 (NNI > 0.8) are indicated as circles and rectangles, respectively.

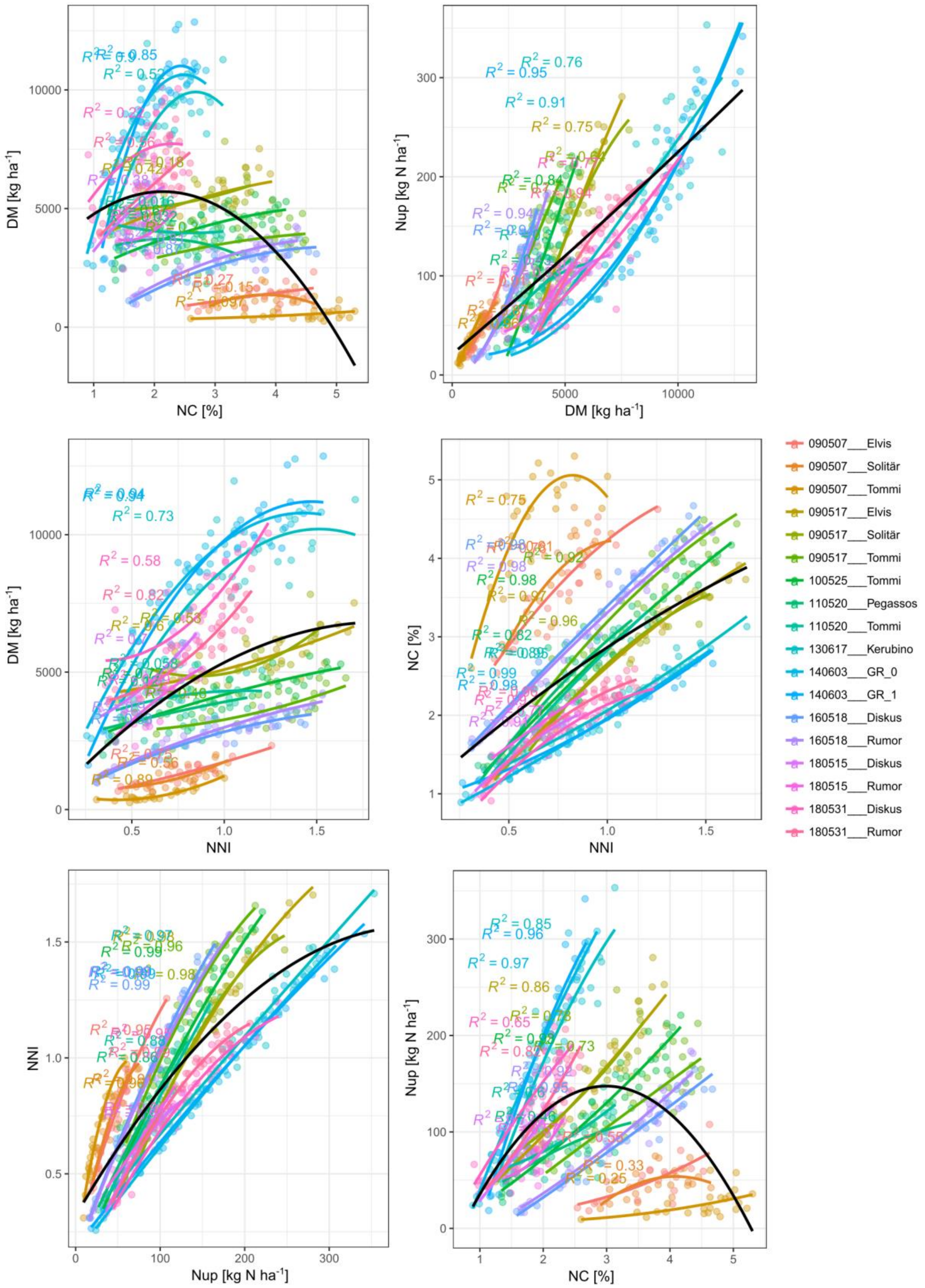

Supplementary Figure 2: Relationships between target traits by measurement dates. Curves indicate best-fit quadratic relationships .

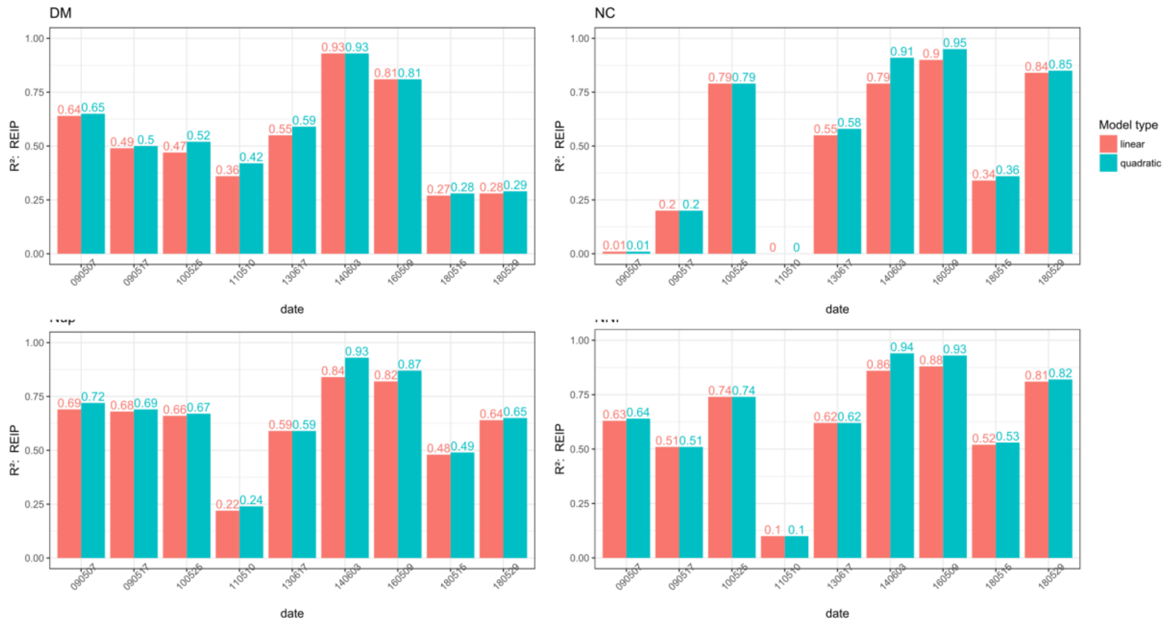

Supplementary Figure 3: Coefficients of determination (R^2) of the REIP within dates across main plot treatments for linear and quadratic relationships.

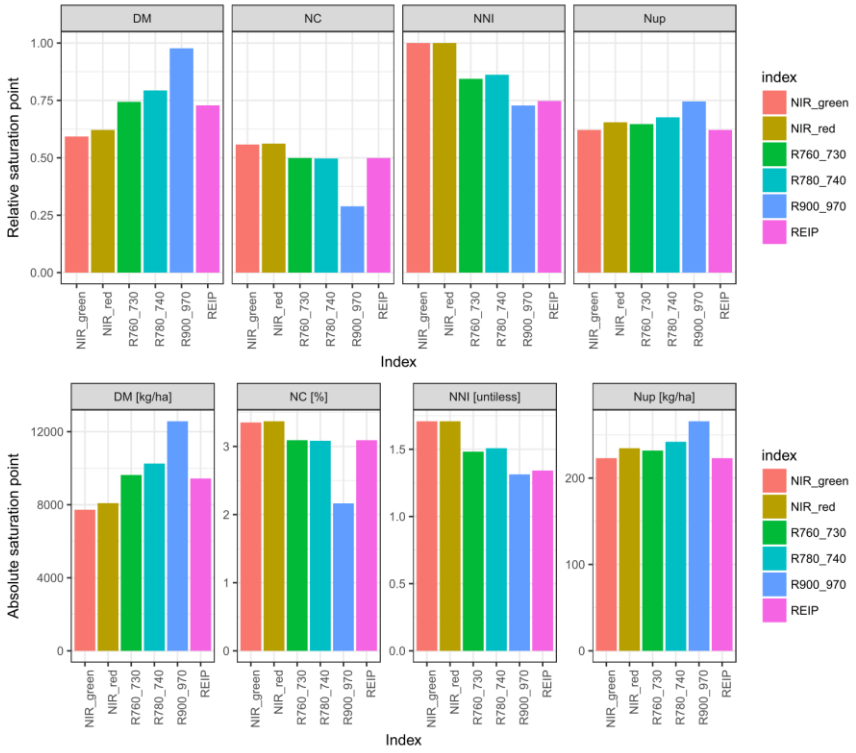

Supplementary Figure 4: Point of saturation (plateau point) relative to the present data range and in absolute values, identified as the first point of non-positive slope between index and target trait.

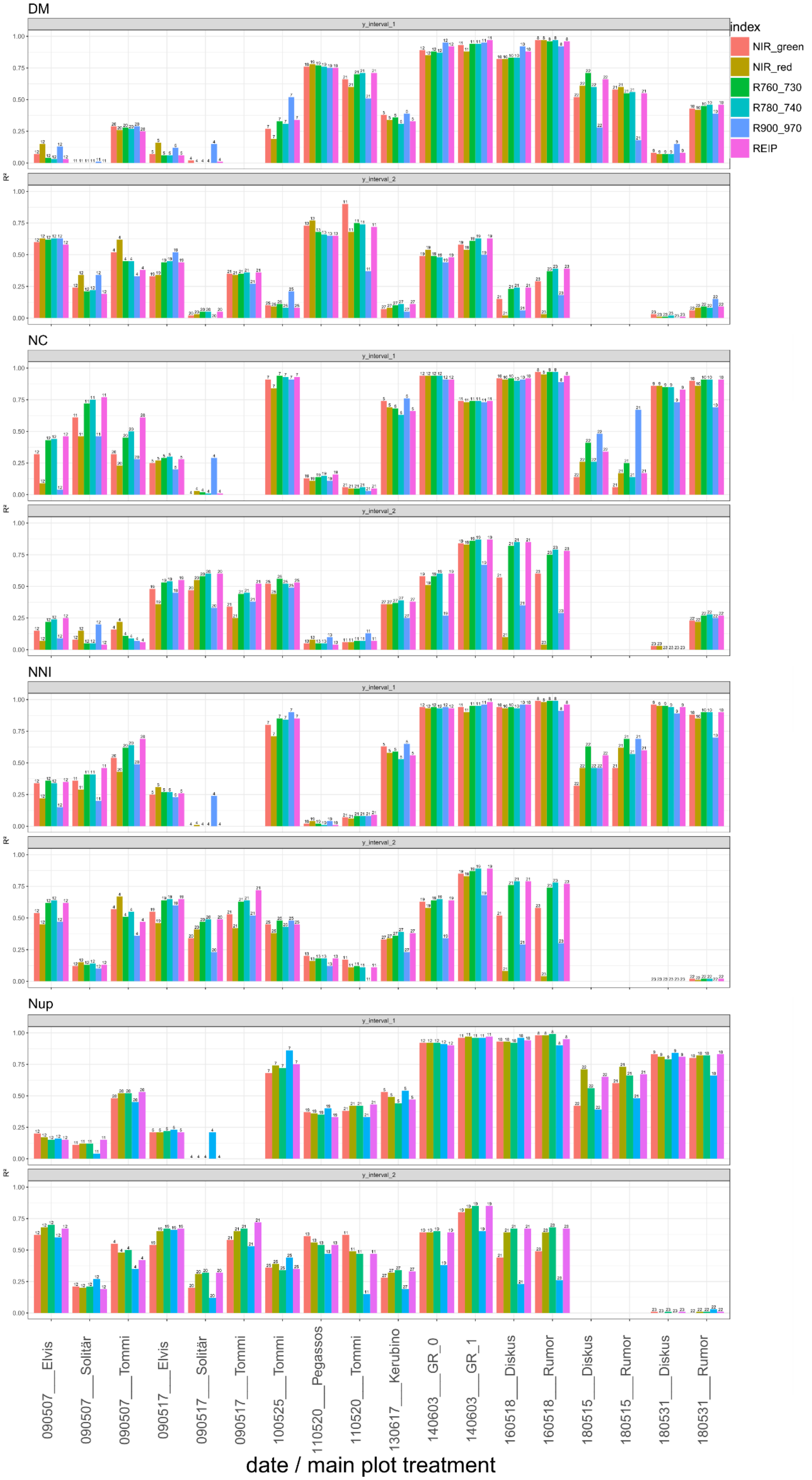
DM
NC
NNI
Nup
y_interval_1
y_interval_2
R²
index
NIR_green
NIR_red
R760_730
R780_740
R900_970
REIP
090507___Elvis
090507___Solitär
090507___Tommi
090517___Elvis
090517___Solitär
090517___Tommi
100525___Tommi
110520___Pegassos
110520___Tommi
130617___Kerubino
140603___GR_0
140603___GR_1
160518___Diskus
160518___Rumor
180515___Diskus
180515___Rumor
180531___Diskus
180531___Rumor
date / main plot treatment

Supplementary Figure 5: R^2-values found from linear regression analysis for both NNI-based data intervals, based on the NNI-threshold 0.8. Black numbers indicate the number of data points (n) included in the intervals.

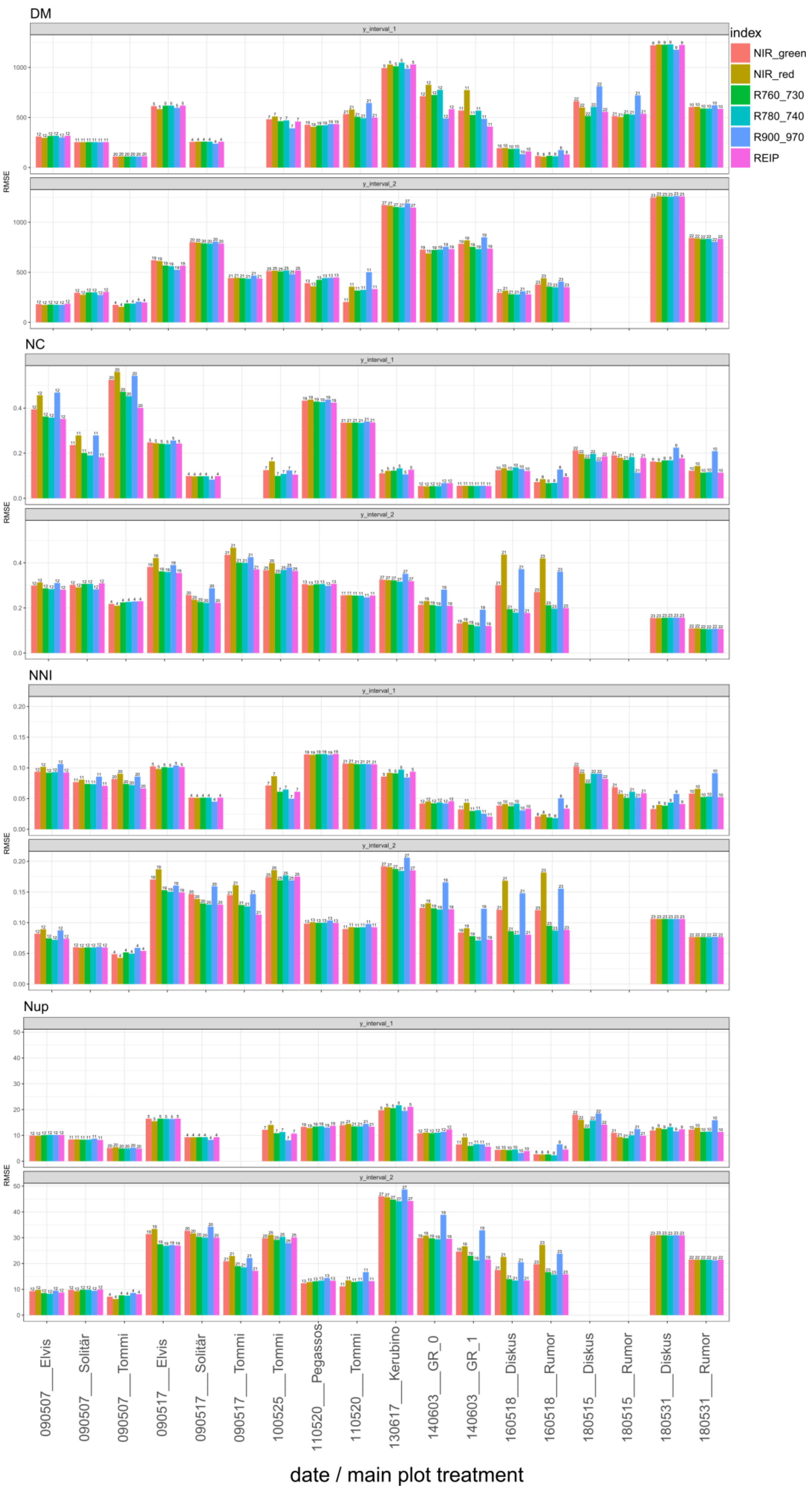
DM
NC
NNI
Nup
y_interval_1
y_interval_2
RMSE
index
NIR_green
NIR_red
R760_730
R780_740
R900_970
REIP
090507___Elvis
090507___Solitär
090507___Tommi
090517___Elvis
090517___Solitär
090517___Tommi
100525___Tommi
110520___Pegassos
110520___Tommi
130617___Kerubino
140603___GR_0
140603___GR_1
160518___Diskus
160518___Rumor
180515___Diskus
180515___Rumor
180531___Diskus
180531___Rumor
date / main plot treatment

Supplementary Figure 6: RMSE-values found from linear regression analysis for both NNI-based data intervals based on the NNI-threshold 0.8 for DM [kg ha^{-1}], NC [%], NNI [unitless] and Nup [kg ha^{-1}]. Black numbers indicate the number of data points (n) included in the intervals.
